# Metabolites profiling, in-vitro and molecular docking studies of five legume seeds for Alzheimer’s disease

**DOI:** 10.1038/s41598-024-68743-7

**Published:** 2024-08-23

**Authors:** Rana M. Ibrahim, Passent M. Abdel-Baki, Osama G. Mohamed, Ahmed A. Al-Karmalawy, Ashootosh Tripathi, Riham A. El-Shiekh

**Affiliations:** 1 Pharmacognosy Department, Faculty of Pharmacy, Cairo University, Kasr-El-Ainy Street, Cairo, 11562 Egypt; 2 Natural Products Discovery Core, Life Sciences Institute, University of Michigan, Ann Arbor, MI 48109 USA; 3 Department of Pharmaceutical Chemistry, Faculty of Pharmacy, Horus University-Egypt, New Damietta, 34518 Egypt; 4 Pharmaceutical Chemistry Department, Faculty of Pharmacy, Ahram Canadian University, 6th of October City, Giza, 12566 Egypt; 5 Department of Medicinal Chemistry, College of Pharmacy, University of Michigan, Ann Arbor, MI 48109 USA

**Keywords:** Legumes, Nutraceutical, Fabaceae seeds, Metabolomics, Docking study, Alzheimer, Plant sciences, Chemistry

## Abstract

Even though legumes are valuable medicinal plants with edible seeds that are extensively consumed worldwide, there is little information available on the metabolic variations between different dietary beans and their influence as potential anti-cholinesterase agents. High-resolution liquid chromatography coupled with mass spectrometry in positive and negative ionization modes combined with multivariate analysis were used to explore differences in the metabolic profiles of five commonly edible seeds, fava bean, black-eyed pea, kidney bean, red lentil, and chickpea. A total of 139 metabolites from various classes were identified including saponins, alkaloids, phenolic acids, iridoids, and terpenes. Chickpea showed the highest antioxidant and anti-cholinesterase effects, followed by kidney beans. Supervised and unsupervised chemometric analysis determined that species could be distinguished by their different discriminatory metabolites. The major metabolic pathways in legumes were also studied. Glycerophospholipid metabolism was the most significantly enriched KEGG pathway. Pearson’s correlation analysis pinpointed 18 metabolites that were positively correlated with the anti-cholinesterase activity. Molecular docking of the biomarkers to the active sites of acetyl- and butyryl-cholinesterase enzymes revealed promising binding scores, validating the correlation results. The present study will add to the metabolomic analysis of legumes and their nutritional value and advocate their inclusion in anti-Alzheimer’s formulations.

## Introduction

Negative psychological and physiological impacts of neurodegenerative disorders have a substantial social and health cost^1^. Among them, is Alzheimer’s disease which is an irreversible neurodegenerative disease in the brain and the main cause of dementia^2^. It is gradually age-related, noted by gradual mental failure, behavioral disturbances, cognitive deficits, speech impairment, and diminished verbal fluency. According to the current epidemiological data, about 50 million people worldwide suffer from Alzheimer’s disease, and these numbers are predicted to triple by 2050^3^. Running treatments have not been effective cures until now; they only alleviate the symptoms and temporarily slow the rate of cognitive progression linked with Alzheimer’s disease. Most of the medicines are approved to act as acetylcholinesterase (AChE) inhibitors (donepezil, rivastigmine, tacrine, and galantamine). However, most of them commonly result in side effects viz*.* hepatotoxicity, diarrhea, insomnia, or sickness^1^. So, therapeutic goals based on natural ingredients were highly recommended. Natural products are capable of reducing the symptoms and slowing the progression of several diseases including Alzheimer’s disease attracting the attention of the scientific field and pharmaceutical industries^4^. They have demonstrated anti-inflammatory, antioxidant, anti-amyloidogenic, and anti-cholinesterase activities including flavonoids, anthocyanins, triterpenes, and alkaloids^1^.

Legumes are an essential part of the diet in several countries worldwide, particularly as a source of dietary protein in developing countries^5^. Recently, interest in legumes has increased because of their beneficial or protective actions on human health. Many studies have reported that frequent consumption of legumes reduces the risk of type 2 diabetes, certain types of cancer, cardiovascular disease, overweight or obesity^6^. These activities could be attributed to the nutritional composition of pulses and their bioactive secondary compounds such as flavonoids, phenolic acids, tannins, and saponins, in addition to others^7,8^. The most consumed legumes (Fabaceae family) are chickpeas (*Cicer arietinum* L.), lentils (*Lens culinaris* Medik), and beans (*Phaseolus vulgaris* L.).

Due to the complicated metabolic nature of plant matrices, untargeted metabolomics is increasingly popular in food analysis and regarded as the preferred technique for metabolic profiling of plant metabolites^9,10^. It helps researchers better comprehend the complexity of these mixtures by providing an objective method for comparing metabolite profiles between groups, enabling us to detect new markers to fight against food fraud^11^.

Over the last few years, metabolomics approaches have been engaged in the field of food sciences as powerful tools to ensure food safety, quality, and traceability^12^. This has brought attention to the need to develop and apply techniques such as metabolomics that make it possible to stay abreast of the new requirements of the food market^12,13^. Metabolomics approaches will allow the industry to analyze food quality and food authentication^12^, and aiding in determining which metabolites are most relevant for certain biological activities^14^. To fully exploit, analyze, and extract valuable information from the vast amounts of chemical data produced, chemometric tools such as principal component analysis (PCA) and partial least squares discriminant analysis (PLS-DA), have become a must^9^. In a nutshell, non-targeted metabolic analysis using UHPLC-Q-TOF-MS in conjunction with chemometrics can be a powerful strategy for detecting multi-class components, finding reliable markers for distinguishing between closely related species, and understanding differences in their biological potential.

Compared to previous LC-MS/MS studies on legume seeds, several studies have used targeted metabolomics analysis^15^ and directed to specific metabolite classes, such as flavonoids^16^, anthocyanins^17^, and saponins^18^ or particular Leguminosae species, such as faba beans^19^, chickpeas^20^, and lentils^21^. However, there is relatively little information available on the full phytochemical composition of numerous legumes utilizing untargeted metabolomic methods^22^.

This study is the first to provide comprehensive metabolite fingerprints of five edible legume seeds (fava beans, black-eyed peas, kidney beans, chickpeas, and lentils) using an untargeted LC-MS-based metabolomics approach combined with chemometrics as a useful tool to assess metabolite heterogeneity and trace the bioactive compounds, contributing to the medicinal significance of these highly consumed seeds in human diets. Furthermore, the Kyoto Encyclopedia of Genes and Genomes (KEGG) enrichment analysis indicated the most significant biochemical metabolic pathways implicated by the identified metabolites, which contributed to the metabolic discrepancy among the samples.

To evaluate the therapeutic efficacy of new sources, some important in-vitro biological assays were performed firstly, acetyl- (AChE) and butyryl-cholinesterase (BuChE)^23–26^ beside antioxidant assays such as [2,2′-azino-bis-3-ethylbenzthiazoline-6-sulfonic acid (ABTS), ferric-reducing antioxidant power (FRAP), and 1,1-diphenyl-2-picrylhydrazyl (DPPH)]^27,28^.

Our study was aimed at searching for more nutraceuticals to prevent and diminish the symptoms of Alzheimer’s disease, where the comprehensive profiles of five commonly used legumes in human diet (*Vicia faba* L. (fava beans, FB), *Vigna unguiculata* (L.) Walp. (black-eyed pea, BP), *Phaseolus vulgaris* L. (common bean, kidney bean, KB), *Lens culinaris* L. (lentils, red lentil, RL), and *Cicer arietinum* L. (chickpea, CP) were investigated using LC/MS-based metabolomics. The antioxidant and anticholinergic effects of the seeds were determined using multiple *in-vitro* assays. The differentially accumulated metabolites and the top enriched metabolic pathways in the studied seeds were annotated. The metabolite-bioactivity correlation was determined using Pearson’s correlation. In addition, molecular docking simulations were used to examine the binding affinities and interactions of the identified compounds with AChE and BuChE active sites. The paper may represent the first comprehensive document of the multi-targeted potential of these legumes applying metabolomics to establish which compounds discriminate between the five studied legumes (*Vicia faba* L. (fava beans, FB), *Vigna unguiculata* (L.) Walp. (black-eyed pea, BP), *Phaseolus vulgaris* L. (common bean, kidney bean, KB), *Lens culinaris* L. (lentils, red lentil, RL), and *Cicer arietinum* L. (chickpea, CP), while assessing their health-promoting potential against Alzheimer’s disease to optimize their use in the food, supplements, and pharmaceutical industries.

## Material and methods

### Chemicals

Trolox, 1,1-diphenyl-2-picrylhydrazyl (DPPH), 2,2′-azino-bis-3-ethylbenzthiazoline-6-sulphonic acid (ABTS), FeCl_3_, Tris-HCl buffer, dithio-bis-(2-nitrobenzoic acid (DTNB), tripyridyl triazine (TPTZ), butyrylthiocholine iodide, DMSO, methanol for HPLC, acetylthiocholine, acetylcholinesterase (AChE) from Electric eel and butyryl-cholinesterase (BuChE) from equine serum were obtained from Sigma Chemical Co. (St. Louis, MO, USA).

### Plant material and extraction

Five seeds of Fabaceae family; *Vicia faba* L. (fava beans, FB), *Vigna unguiculata* (L.) Walp. (black-eyed pea, BP), *Phaseolus vulgaris* L. (common bean, kidney bean, KB), *Lens culinaris* L. (lentils, red lentil, RL), and *Cicer arietinum* L. (chickpea, CP) were obtained from Agricultural Research Center, Giza, Egypt, in February 2022. The plant collection complies with relevant institutional, national, and international guidelines and legislation. Samples were kept in the Herbarium of the Pharmacognosy Department, Faculty of Pharmacy, Cairo University. Voucher specimen numbers were 23-9-2019 I-V. The dried seeds (100 gm) were ground by a coffee grinder (Black & Decker SmartGrind Model CBG5, The Black & Decker Corporation, USA) to a fine powder that would pass through a 0.5 mm screen. The powder of each studied plant was extracted with methanol (500 mL × 4) till exhaustion. Solvents were removed with a rotary evaporator at 50 ℃ to obtain the extracts in nearly an hour. Ten mg of the solid extracts were dissolved in 1 mL of methanol, centrifugated at 13000 g for 10 min, filtered (syringe nylon filter 0.22 µm pore diameter), and kept at −20 ℃ till further LC-MS analysis. For each sample, 3 biological replicates were extracted in parallel under the same conditions. For biological analysis, 20 mg of dried methanolic extracts were dissolved in DMSO.

### Antioxidant activity

Antioxidant activities of both samples were performed using ferric-reducing antioxidant power (FRAP)^29^, 1,1-diphenyl-2-picrylhydrazyl (DPPH)^30^, and 2,2′-azino-bis-3-ethylbenzthiazoline-6-sulphonic acid (ABTS)^29^ antioxidant capacities.

#### FRAP

The reaction mixture consists of TPTZ solution (0.25 mL; 10 mM) in HCl (40 mM), FeCl_3_ (0.25 mL; 20 mM), the samples at different concentrations, and acetate buffer (2.5 mL; 300 mM, pH 3.6). The FRAP reagent (170 μL), and the tested samples (20 μL) were mixed in a 96-well plate and incubated for 30 min in a dark place. Following incubation for 30 min, the maximum absorbance was measured at 593 nm.

#### DPPH

DPPH was prepared in a concentration of 0.04 g% in methanol. All samples were prepared in MeOH. Then, 20 μL of the different concentrations of the samples were added to 200 µl of 2,2-diphenyl-1-picrylhydrazyl (DPPH), in a 96-well plate. The absorbance was measured at 492 nm after incubating for 30 min in the dark at room temperature.

#### ABTS

A working solution of ABTS^·+^ radicals was performed by the reaction between ABTS (7 mM) and potassium persulfate (2.5 mM) at a 1:1 (*v/v*) ratio. The reaction mixture was stocked in the dark at room temperature for 15 h. This solution was diluted with ethanol until an absorbance of 0.70 was recorded at 734 nm. The different concentrations of the samples (20 μL) were added and ABTS solution (200 μL). The mixture was incubated in the dark for 30 min, and the absorbance was calculated at 734 nm.

For all the assays, Trolox was used as a standard (10–1000 μg mL^−1^), and the experiments were performed in triplicate. The following dilutions from the different samples were prepared (7.8125–1000 μg mL^−1^). All the results were expressed as **IC**_**50**_, calculated as follows: **IC**_**50**_** = [(A**_**c**_** – A**_**s**_**) / A**_**c**_**] × 100.** Where A_c_ was the absorbance of the control (Reagent solution without test sample) and A_s_ was the absorbance of the sample [Reagent solution + sample (extract/standard)].

### Cholinesterase inhibitory activity determination

Cholinesterase inhibitory activities (AChE, and BuChE) were performed as mentioned by the standard technique^31–33^. In brief, 170 μL of tris-HCl buffer (200 mM, pH 7.5) was included followed by 20 μL of tested samples (250–7.8125 μg mL^−1^), then 20 μL of the enzyme solution (0.1 U mL^−1^). After incubation of 10 min at 25 ℃, 40 μL of DTNB and 20 μL of the substrate (1.11 mM) were added. Butyrylthiocholine iodide and acetylthiocholine were exploited as substrates in BuChE and AChE assays, respectively, where DTNB behaved as an indicator. All samples were prepared in methanol. The intensity of the developed color was measured at 405 nm using a microplate reader (reading A) and control without the inhibitor was measured (reading B). Blank assays were run by replacing the enzyme (20 μL) with buffer and their absorbances were documented for correction of the spontaneous lysis of the indicator or inherent color of the inhibitor. Linear regression was used for the calculation of the IC_50_ (50% inhibitory concentration). % Inhibition = [1 − (corrected A (Reading A- Blank)/corrected B (Reading B- Blank))] × 100. Donepezil was used as standard (0.48–15.625 μg mL^−1^).

### UHPLC analysis and data processing

Ultra-high-performance liquid chromatograms (UHPLC) were obtained on an Agilent LC-MS system composed of an Agilent 1290 Infinity II UHPLC coupled to an Agilent 6545 ESI-Q-TOF-MS in both negative and positive modes adopted the previously described method^25,34^.

Raw data of the three biological replicates acquired from the UHPLC-QTOF-MS/MS was first converted to the mzML format by msConvert software and then processed by using the mzmine 3 software (http://mzmine.github.io/) for peak extraction^35^. Mass ion peaks were isolated with a centroid detector threshold with the noise level set to 1.0 × 10^2^ and an MS level of 1. Chromatogram builder was used with a minimum time span set to 0.1 min, and the minimum height and *m/z* tolerance to 1 × 10^4^ (positive mode), 5 × 10^3^ (negative mode), and 0.001 *m/z* or 5.0 ppm, respectively. Chromatogram deconvolution was performed using a baseline cut-off algorithm with minimum peak height: 1 × 10^4^ (positive mode), 5 × 10^3^ (negative mode), peak duration range (0–0.4 min), and baseline level: 5 × 10^2^. The separated peaks were then deisotoped using the function of isotopic peaks grouper (*m/z* tolerance: 0.001 *m/z* or 5.0 ppm, retention time tolerance: 0.2 absolute (min), maximum charge: 2, and representative isotope: most intense). The parameters for data filtering, gap-filling, and the retention time normalizer were set to *m/z* tolerance: 0.001 *m/z* or 5.0 ppm, retention time tolerance: 0.2 absolute (min), and minimum standard intensity: 1 × 10^4^ (positive mode), 5 × 10^3^ (negative mode). The peak lists were all aligned using the join aligner with *m/z* tolerance: 0.001 *m/z* or 5.0 ppm, weight for *m/z*: 20, retention time tolerance: 5.0 relative (%), weight for RT: 20. An adduct search was performed for Na^+^, K^+^, NH^+4^, formate, and ACN^+^ (RT tolerance: 0.2 absolute (min), *m/z* tolerance: 0.001 *m/z* or 5.0 ppm, max relative adduct peak height: 50%). The processed data was then subjected to formula prediction by selecting atoms C, H, N, O, and any other elements and adjusting parameters with heuristics element count with all three sub-options to get the isotope pattern filter working with all features with isotope peaks.

### Statistical analysis

The obtained CSV file, including the normalized peak areas and identities (retention time (tr), mass-to-charge (*m/z*), molecular formula, and name) of the 130 metabolites identified in the three replicates of the five samples were exported to the online platform MetaboAnalyst 5 (https://www.metaboanalyst.ca/MetaboAnalyst/ModuleView.xhtml) for further chemometric analysis, including Hierarchical clustering heatmap (HCA), Volcano plot, principal component analysis (PCA), and partial least squares discriminant analysis (PLS-DA). Moreover, the variable importance in the projection (VIP) values was calculated to identify the metabolites that significantly differentiated between the five legume samples. For statistical significance, one-way ANOVA and Tukey’s HSD post-hoc analysis with *p*-value threshold less than 0.05 were selected. Furthermore, the biological significance of the identified metabolites was declared through the metabolite set enrichment analysis using the Kyoto Encyclopedia of Genes and Genomes (KEGG) pathway analysis. Representation of the metabolic pathways is displayed according to their significance arranged by p-values (y-axis, pathway enrichment analysis) or pathway impact (x-axis, pathway topology analysis). The dot color is based on the p-value and its size is defined by the pathway impact values calculated from the matched metabolites. The biomarker compounds correlated to the anti-cholinesterase activity were determined using Pearson’s correlations.

For biological studies, data are presented as mean ± standard deviation (SD) and the comparison is made by the one-way ANOVA (variance analysis) with post-hoc Tukey HSD test for multiple comparisons at a significance level of 0.05.

### Docking study

#### Two molecular docking studies

The identified compounds were docked against both AChE and BuChE target enzymes and compared to their co-crystals as well. This was performed to clarify the anti-Alzheimer effects of the investigated compounds at the molecular levels using in silico drug design tools^36,37^. The chemical structures of the tested metabolites were created in ChemDraw and transferred to the working window for energy minimization and correction^38^. The target receptors (AChE and BuChE) were obtained from the Protein Data Bank (https://www.rcsb.org/structure/4EY7 and https://www.rcsb.org/structure/8CGO), respectively. Each target was prepared for the docking step through the correction of errors, 3D hydrogenation, and energy minimization steps^39,40^. The previously prepared compounds were inserted in two different databases with the co-crystal of each target receptor as a reference^41^. The superior candidates were selected according to their binding modes, binding scores, and RMSD (Root Mean Square Deviation)^42^. Besides, two validation processes were carried out by redocking the co-crystal of each target receptor within its binding site which showed similar binding modes to the native co-crystals and low RMSD values (< 2 Å) as well^40^.

## Results and Discussion

### In-vitro biological activities

Antioxidant and anti-Alzheimer activities of the tested samples are summarized in Table 1. Generally, the studied legume methanolic extracts showed good antioxidant and anti-Alzheimer activities with chickpea seeds exerting almost the best results as revealed by their lower IC_50_ values which were close to standard (Table 1). In the antioxidant assays (ABTS, FRAP, and DPPH), all the samples showed better results than the standard (Trolox). Whereas, in AChE assay, CP showed the highest activity among other tested samples as compared to the reference drug (Donepezil), followed by KB sample (Table 1). In BuChE assay, the studied legume methanolic extracts showed strong activity^43^ as compared to the reference drug, with CP and KB exhibiting the best results.

**Table 1:** In-vitro antioxidant and anti-Alzheimer activities of five seeds of Fabaceae family.

Samples	ABTS	FRAP	DPPH	AChE	BuChE
	IC_50_ (μg/mL), Mean ± SD				
FB	20.22 ± 1.45^b^	33.01 ± 3.1^c^	14.19 ± 2.7^a^	6.07 ± 0.49^d^	10.12 ± 0.88^c^
BP	17 ± 1.46^ab^	23.45 ± 1.56^b^	13.09 ± 1.13^a^	12.10 ± 2.5^e^	10.08 ± 2.06^c^
KB	22.11 ± 1.81^b^	29.31 ± 1.5^c^	18.54 ± 1.5^b^	5.5 ± 0.42^c^	7.03 ± 0.6^b^
RL	14.01 ± 2.11^a^	25.31 ± 1.75^b^	16.19 ± 0.78^a^	6.34 ± 0.56^d^	10.1 ± 0.27^c^
CP	19.17 ± 3.15^ab^	13.81 ± 2.5^a^	12.5 ± 2.2^a^	2.66 ± 0.33^b^	8.02 ± 0.35^b^
Trolox	69.17 ± 3.15^c^	207.2 ± 8.19^d^	20.5 ± 1.2^b^	–	–
Donepezil				0.23 ± 0.03^a^	0.3 ± 0.0033^a^

Identical letters per column indicate no significant differences, p ≤ 0.05.

IC_50_: strong < 5 μg/mL; moderate: 5–20 μg/mL; weak: 20–100 μg/mL and not active > 100 μg/mL.

Legumes are a source of the biologically active compounds classified as phenolic acids, flavanols, flavan-3-ols, tocopherols, anthocyanins/anthocyanidins, vitamin C, and condensed tannins/proanthocyanidins, which are responsible for their antioxidant activity^44,45^. P. Siddhuraju, and K. Becker (2007) proved the antioxidant activity of BP using tests such as DPPH, ABTS, and FRAP, and the results were significantly lower than the reference drugs (quercetin, butylated hydroxyanisole, and Trolox)^46^. It is worth highlighting that the studied legume methanolic extracts were investigated for the first time as ChE inhibitors.

### UHPLC/MS metabolite profiling

Legumes are a habitual part of the diet in several countries worldwide, especially as a source of phytochemicals and nutrients. The methanolic extracts of five commonly used seeds in Fabaceae family were analyzed using UHPLC-Q-TOF-MS in both negative and positive ionization modes to obtain their comprehensive profiles and correlate their metabolites with anticholinergic potential. The representative base peak chromatograms of the samples are demonstrated in Supplementary Materials Fig. S1. The identification was based on several databases the MassBank of North America (https://mona.fiehnlab.ucdavis.edu/https://mona.fiehnlab.ucdavis.edu/), LOTUS: Natural Products Online Database (https://lotus.naturalproducts.net/), Human Metabolome Database (http://www.hmdb.ca/), KEGG (https://www.genome.jp/kegg/kegg1.html), LipidMaps (https://www.lipidmaps.org/), and MassBank of North America (https://mona.fiehnlab.ucdavis.edu/). In addition, a literature review of the reported chemical constituents in Fabaceae family^22,47–51^. In total, 139 compounds were tentatively identified using MS^2^ level of confidence^52,53^ and characterized by their retention time (tr), accurate molecular monoisotopic mass, and MS/MS spectra in the five extracts. The details on their accurate masses, molecular formulas, retention time, tentative identification, and chemical class are shown in Table S1, Fig. S1. These compounds can be classified into several chemical classes, including, amino acids and amine derivatives, carbohydrates, phenolic acids, alkaloids, anthocyanins, saponins, fatty acids and fatty acyls, flavonoids, isoflavonoids, lignans, iridoids, stilbenes, sterols and terpenes, benzophenones, and phospholipids. To the best of our knowledge, this is the first comparative metabolite profiling of the five seeds coupled with chemometrics and computational analysis, which is traced to provide chemical-based evidence for their differential biological effect on Alzheimer’s key enzymes.

#### Amino acids and sugars

Eight amino acids were identified including choline, tyramine, isovaline, pipecolic acid, Glu-Tyr, isoleucine, Glu-Leu, and Glu-Phe, additionally to one amine derivative, thermospermine. All were detected in positive ionization mode. Amino acids have a neuroprotective role by improving cognitive function and memory performance^54^, ameliorating the injury-induced cognitive impairment to prevent the progression of Alzheimer^55^. Five sugars were identified in the negative mode *i.e.,* stachyose, galactosyl ciceritol, raffinose, sucrose, and dehydro-ciceritol (Table S1). Sugars showed a neuroprotective effect through the elevation of glutathione level^56^ in previous works.

#### Flavonoids, benzophenone, and phenolic acid

Phenolics have been demonstrated to have neuroprotective roles by boosting neuronal survival, tissue perfusion, cerebral blood flow, and reducing ischemic-related apoptosis^57,58^. Flavonoid subclasses, mainly flavonols and isoflavones, are widely distributed in Fabaceae and exhibit antioxidant and anti-inflammatory activities. Four kaempferol and two quercetin derivatives were identified by fragment ions at *m/z* 285 for kaempferol, and 301 for quercetin^59^. Further fragment ions were at *m/z* 178 (C_8_H_3_O_5_) (1,2A-) and 151 (C_7_H_3_O_4_) (1,3A-), due to the release after the retro Diels-Alder fission and retrocyclization, respectively^60^. Two flavan-3-ols; dihydro-(epi)catechin-diglucoside and catechin-3-*O*-glucopyranoside were also detected as previously reported in the Fabaceae family^61^. Five isoflavonoids; sissotrin, 6''-*O*-malonylgenistin, genistein 7-*O*-apiofuranosyl-(1 → 6)-glucoside, glabridin, and genistin were detected with characteristic ions related to the fission at 0,3B-, such as fragment ions with *m/z* values above 133.0294 (even ion) (C_8_H_5_O_2_) and 132.0217 (odd ion) (C_8_H_4_O_2_)^60^. Caffeic acid derivatives were detected in the investigated samples at early retention time and characteristic fragmentation pattern of caffeic acid at *m*/*z* 341, 191, 179, and 161^62^. Trihydroxy methoxybenzophenone was detected with main fragments at *m/z* 245, 229, 213, and 211 due to the successive losses of hydroxyl groups^63^ (Table S1).

#### Lignans and irridoids

To date, all the available data suggest that the iridoids are a family of natural lipophile chemicals with the features of endogenous neurotrophic factors, which could be considered promising leads for the treatment of neurological disorders^64^, mainly through their anti-inflammatory effects^65^. Furthermore, lignans are among the compounds revealing nitric oxide (NO)-inhibiting activity, where NO is one of the most studied promoters of neuroinflammation^66^. Also, lignans showed inhibitory effects on beta-amyloid (Aβ_1-42_) aggregation^67^.

Oleuropein and its aglycon were identified as secoiridoids and showed main fragments at *m/z* 377 (M − 162), 345 (M − 194), 307 (M − 232), and *m/z* 275 (M − 264)^68^ **(**Fig. S2**)**.

Three lignans syringaresinol, secoisolariciresinol diglucoside, and its isomer were detected and showed main fragments at *m/z* 179 and 219 which are fragment ions formed through benzylic cleavage and containing one aromatic ring and one hydroxy group (*m/z* 179) or one hydroxy and one methoxy group (*m/z* 219)^69^.

#### Saponins, sterols, and terpenes

Saponins have been demonstrated to have neuroprotective activity by improving neuronal synthesis, and synaptic activity^70^. Fourten soyasponins; phaseoside IV, soyasapogenol C, phaseoside I, soyasaponin Bd, soyasaponin Be, soyasaponin Bb, soyasaponin III, kaikasaponin III, soyasaponin alphag II, dehydro-soyasaponin I, soyasaponin alphag I, soyasapogenol E, dihydro-soyasaponin Ba, and soyasaponin II were detected.

A set of peaks belonging to group B of soyasaponins, namely soyasaponins Ba, Bb, Bd, and Be, showed mass fragments at *m/z* 797, 617, 599, and 581 due to successive losses of a hexose moiety along with the neutral loss of one and two molecules of water, respectively. The product ion at *m/z* 459 is generated from the cleavage of the glycosidic bond and formation of soyasapogenol B aglycone; the ensuing product ions at *m/z* 441, 423, and 405 are most likely due to one, two, and three water loss, respectively^71^. Four sterols dihydro-dihydroxy megastigmadien-one-[apiosyl-glucoside], coumestrol acetate, β-sitosterol-3-*O*-glucoside, and stigmastenone were also detected as non-polar metabolites in addition to gibberellin A8 (diterpenes), characteristic to family Fabaceae.

#### Alkaloids, anthocyanins, and stilbene

Alkaloids are a broad class of naturally occurring compounds that have the potential to impact the central nervous system, regulating several body processes and behavior. They are also regarded as cholinesterase inhibitors that enhance memory functions^70^. Two glycoalkaloids were identified in positive ionization mode (Table S1). Fragmentation of β-chaconine (solanidine-glucose-rhamnose) showed fragment ions at *m*/*z* 559 and 397 due to the loss of rhamnose and glucose moieties, respectively^72^. Fragments at *m/z* 204 and 150 are also diagnostic of fragment formulas C_14_H_22_N and C_10_H_16_N, respectively^73^. *N*, *N*′-Diferuloyl-putrescine has antioxidative and skin-whitening activities^74^. It was detected in our profile with main fragments at *m/z* 177, 145, and 117^75^.

Anthocyanins are natural pigments commonly detected in some fruits and vegetables. The most common anthocyanidins in foods are cyanidin, peonidin, pelargonidin, petunidin, malvidin, and delphinidin. Anthocyanins have been documented in several clinical studies as being effective in preventing different disorders such as cardiovascular and neurological disorders^76^. Three anthocyanins were detected in positive ionization mode namely, pelargonidin-3-*O*-glucoside, prodelphinidin B3, and procyanidin dimer B7. Prodelphinidin dimer (prodelphinidin B3) consisting of (epi) gallocatechin—(epi)catechin (*m*/*z* 593.1305) was tentatively identified. The MS^2^ spectrum of this sequence produced ions at *m*/*z* 467, 425, 407, and 289, for heterolytic ring fission of the C ring with a characteristic loss of 126 Da, retro-Diels-Alder fragmentation with a neutral loss of 152 Da, followed by the loss of a water molecule unit (−18 Da), and quinone methide-fission of the inter-flavan bond producing a distinctive loss of 288 Da, respectively^77^. Procyanidin dimer (procyanidins B1, B2, or C1) (*m*/*z* 577.1342) was detected and confirmed by MS^2^ at *m*/*z* 451, 425, 407 and 289^77^. The *m*/*z* 451 was attributed to heterolytic ring fission of the C ring with a characteristic loss of 126 Da. The ion at *m*/*z* 425 was due to retro-Diels-Alder fragmentation with a neutral loss of 152 Da, then the loss of a water molecule unit (−18 Da) at *m*/*z* 407 [M-H − 152 − 18]^−^. The ion at *m*/*z* 289 was due to quinone methide-fission of the interflavan bond producing a distinctive loss of 288 Da. Additionally, pelargonidin-3-*O*-glucoside was identified with the main fragment at *m/z* 271, which corresponded to the pelargonidin aglycon, due to the loss of glucose moiety^78^.

Piceid (resveratrol glucoside) was also detected as stilbenoids with main fragments at *m/z* 269, 241, and 299^77^.

#### Fatty acids and lipids

Polyunsaturated fatty acids can alleviate the cognitive deficits of Alzheimer’s by limiting amyloid polymerization in neuronal cells^79^. The identified fatty acids (Table S1) were malyngic acid, 10-oxo-nonadecanoic acid, hydroxy-tetracosanoic acid, 16-hydroxypalmitic acid, linolenic acid, linoleic acid, octadecadien-1-ol, and β-acetoxyolean-en-oic acid. In addition to, two fatty acyl compounds (tuberonic acid glucoside I, and tuberonic acid glucoside II) and one fatty acid derivative (coumaroyl-caffeoyl-palmitic acid derivative).

Furthermore, the LC-MS analysis revealed the presence of 64 lipids from various classes (Table S1). Each is distinguished by a distinct polar moiety (“head group”) and differs on the inside by the numerous contributing fatty acids with varying degrees of unsaturation and chain length, resulting in distinct fragmentation patterns for each lipid type. In the MS/MS spectra, the presence of carboxylate ions [RCOO]^−^ identifies the particular fatty acids esterified on the glycerol skeleton, *e.g.*, *m/z* 281 for octadecenoic acid (18:1), 279 for octadecadienoic (18:2), 277 for octadecatrienoic (18:3), and 255 for hexadecanoic (16:0) acids, where the esterification position can be determined through the relative intensities of both fatty acid ions (the higher abundant peak assigned for the ion at *sn-2*)^80^. Phospholipids could improve cognitive performance, and exhibit a neuroprotective effect against Alzheimer’s by inhibiting amyloid beta deposition in neural cells^81^.

Our analysis revealed the presence of various chemical classes in the analyzed extracts, confirming the enrichment of these seeds in multifunctional nutraceuticals against Alzheimer’s disease. In consequence, the multivariate data analyses were applied based on the tentatively identified metabolites and their corresponding peak areas to get current insights into the chemical heterogeneity between the Fabaceae seeds and to correlate the key bioactive compounds to the anti-cholinesterase activity of the extracts.

### Metabolite profiles comparison and differentiating metabolites analysis

**Figure 1:**
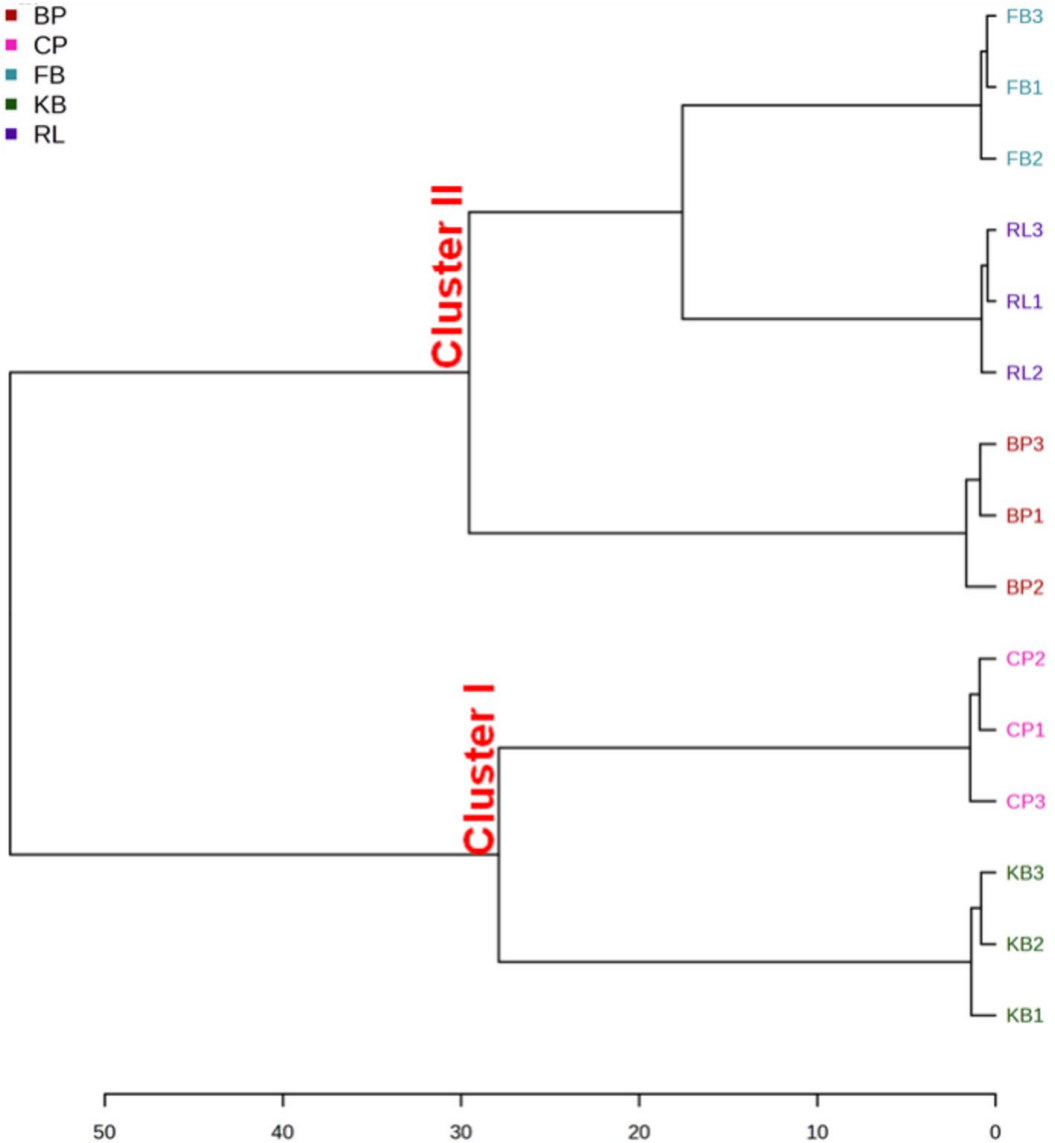
Hierarchical cluster analysis (HCA) dendrogram of the five analyzed legumes based on cluster analysis of mass spectrometric biochemical profiles.

Multivariate statistics were used to further assess the difference in the metabolic profiles among the five legume samples. The unsupervised HCA analysis was performed using the mass data of the identified compounds (Table S1) to classify the five legumes based on the relative differences in the accumulation of secondary metabolites and to identify the holistic discrepancy and similarity in their metabolic profiles in an untargeted and throughput manner. The HCA results shown in Fig. 1 demonstrated that the five legumes were clearly divided into two clusters: KB and CP samples shared comparable metabolic profiles, forming cluster I, whereas FB, RL, and BP samples had distinct chemical composition and/or component levels, constituting cluster II. It is worth noting that within cluster II, FB and RL samples showed higher chemical similarity when compared to BP sample.

**Figure 2:**
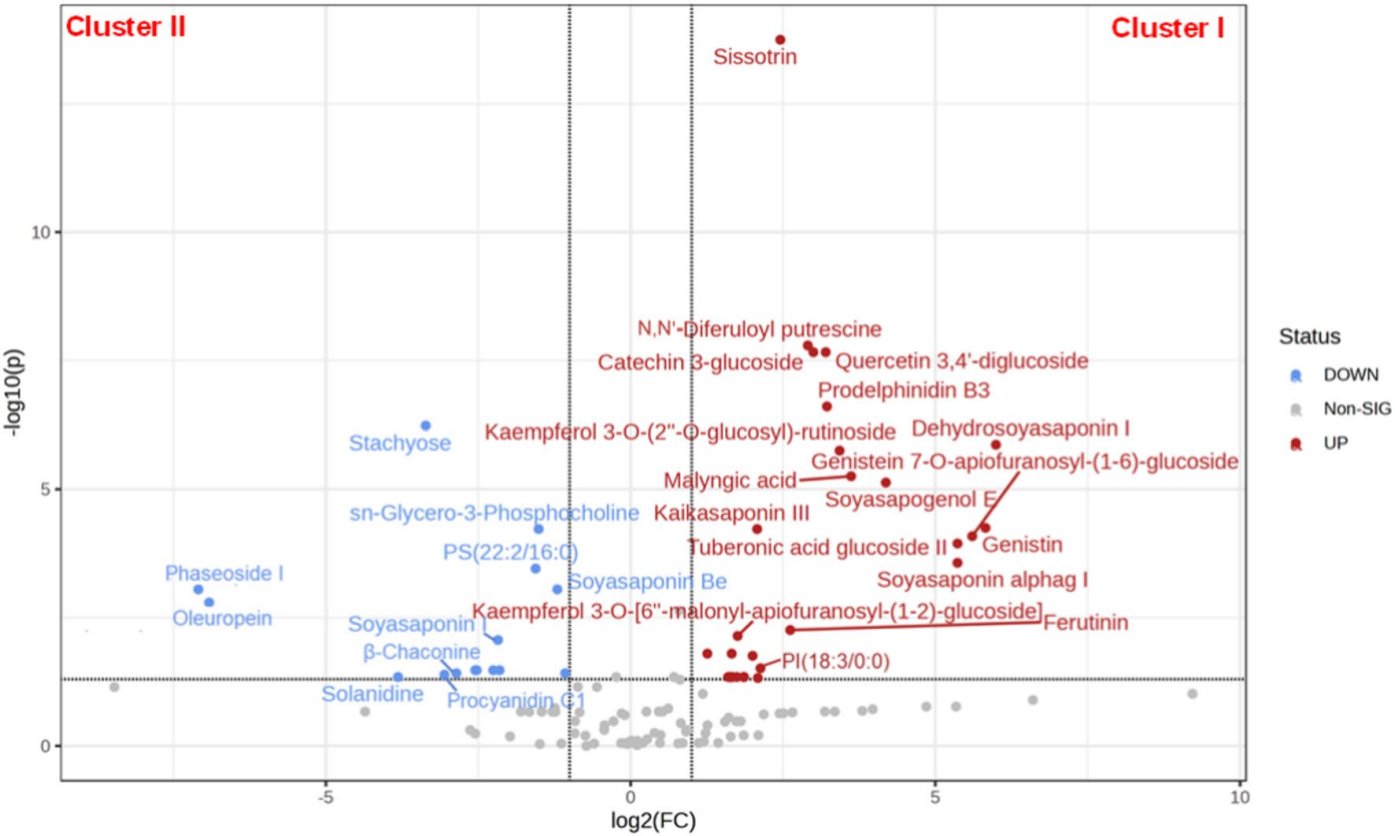
Volcano plot of differential metabolites of cluster I (KB, CP) *vs.* cluster II (FB, RL, BP). The fold-change value (FC) for each differential metabolite was transformed as Log_2_, and the corresponding p-value was transformed as − Log_10_.

After that, a volcano plot (Fig. 2) was used to obtain further insight into the metabolic differences between these two clusters and to determine the differently accumulated compounds and their expression levels. A fold-change (FC) score ≥ 2 or ≤ 0.5 among the identified metabolites with *p*-value < 0.05 was used as an identification criterion. There were 46 differential accumulated metabolites (31 upregulated and 15 downregulated) between cluster I (KB and CP) and cluster II (FB, RL, and BP) samples. Consequentially, the discriminating metabolites colored red were substantially higher in cluster I while being lower in cluster II (FC ≥ 2.0), whereas those colored blue were significantly lower in cluster I but higher in cluster II (FC ≤ 0.5) (Fig. 2). These metabolites mainly comprised saponins, flavonoids, isoflavonoids, alkaloids, phenolic acid derivatives, phospholipids, and procyanidins, as well as sugars, fatty acyls and acids.

**Figure 3:**
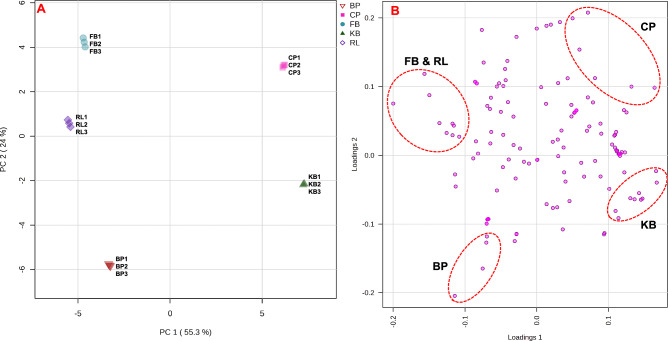
Principal component analysis (PCA) scores plot (**A**) and loadings plot visualization (**B**) using the identified metabolites by LC/MS analysis of the five legume samples (n = 3).

Further, PCA modeling was performed to provide a general visual separation of all of the samples. At 95% confidence limit, two principal components described the positions of the distinct metabolome clusters with PC1/PC2 accounting for 79.3% of the variance in metabolic profiles of the analyzed extracts. In the PCA scores plot (Fig. 3A), the 3 biological replicates of each sample were coincidently grouped together affirming the extraction method consistency, as well as the data analysis stability and repeatability. It was noticeable that the metabolic profiles of CP and KB extracts are grouped on the far-right side of the plot (positive PC1 values) and are fairly distinctive and separated from the other three samples (FB, RL, and PB), located at the left side of the plot (negative PC1 values). The results were similar to the HCA model, with the five samples grouped into two distinct areas in the plot. Examination of the loading plot (Fig. 3B) revealed that MS variables contributed mainly to the separation of CP in the score plot are genistein 7-O-apiofuranosyl-(1 → 6)-glucoside, tuberonic acid glucoside II, trihydroxy methoxybenzophenone, sissotrin, PC (18:1/18:3), and kaikasaponin III. While, those of KB samples are soyasapogenol E, PC (18:2/18:3), dihydro-(epi)catechin-diglucoside, malyngic acid, kaempferol 3-[galactosyl-(1 → 6)-glucoside] 7-[rhamnosyl-(1 → 3)-rhamnoside], dehydro-soyasaponin I, ferutinin, LysoPG(18:2), and LPC(18:0). The signals of secoisolariciresinol diglucoside II, soyasaponin I, solanidine, and PE (18:1/18:2) were the major variables contributing to BP sample’ discrimination. Regarding FB and RL samples, nine metabolites, hydroxy ferutinin, soyasaponin Be, oleuropein aglycone, procyanidin C1, LysoPG(16:0/0:0), PC(20:2/14:1), isoleucine, kaempferol 3-O-[6''-malonyl-apiofuranosyl-(1 → 2)-glucoside] glucoside, and PE(18:2/18:3), accounted for their segregation in the PCA scores plot.

**Figure 4:**
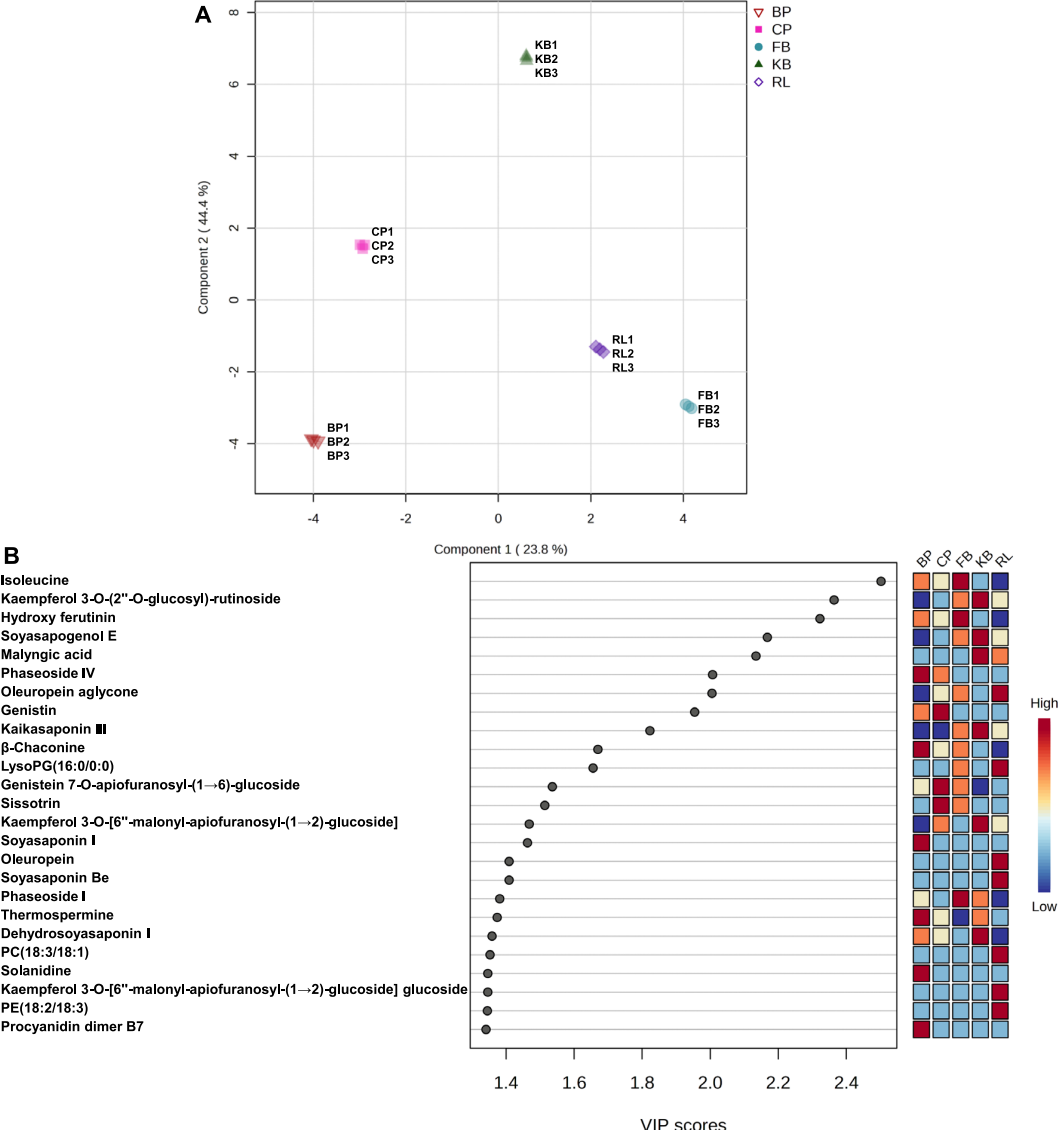
Partial least squares discriminant analysis (PLS-DA) scores plot (**A**) and the variable importance in projection (VIP) score showing the top 25 differential metabolites (VIP scores > 1) (**B**) in the methanolic extracts of the five analyzed legumes.

Unlike PCA, PLS-DA is a supervised multivariate analysis method that can maximize the differences between different groups by using partial least squares regression to model the relationship between metabolite expression and sample class to achieve modeling prediction of the studied samples. Therefore, five-class PLS-DA model (Fig. 4) was used to identify the metabolites that were responsible for the observed separation in PCA. High predictability (Q^2^) and strong goodness of fit (R^2^X, R^2^Y) of the PLS-DA model were observed (Q^2^ = 0.91, R^2^X = 0.73, R^2^Y = 0.93). The fivefold CV-ANOVA and permutation of the cross-validation test (20 iterations) revealed great predictability and goodness of fit of the constructed PLS-DA model (Fig. S3). As can be observed in the PLS-DA scores plot (Fig. 4A), the five legumes were clearly separated from each other and the variable importance in the projection (VIP) value of the first principal component of the PLS-DA model was used at p < 0.05 to find the unique chemical markers for each sample (Fig. 4B) metabolites. Accordingly, the useful markers of BP sample are phaseoside IV, *β*-chaconine, soyasaponin I, thermospermine, and solanidine. Likewise, CP extract was enriched in genistein 7-O-apiofuranosyl-(1 → 6)-glucoside, sissotrin, and genistin. In contrast, FB sample was characterized by a high abundance of phaseoside I, hydroxy ferutinin, and isoleucine. On the other hand, dehydro-soyasaponin I, kaempferol 3-O-[6''-malonyl-apiofuranosyl-(1 → 2)-glucoside], kaikasaponin III, soyasapogenol E, malyngic acid, and kaempferol 3-O-(2''-O-glucosyl)-rutinoside are enriched in KB extract. Finally, RL sample demonstrated higher levels of kaempferol 3-O-[6''-malonyl-apiofuranosyl-(1 → 2)-glucoside] glucoside, PE(18:2/18:3), PC(18:3/18:1), oleuropein, soyasaponin Be, LysoPG(16:0/0:0), and oleuropein aglycone.

### Metabolic pathway enrichment analysis based on KEGG database

**Figure 5:**
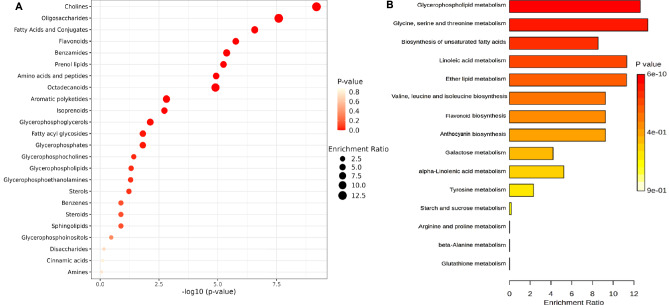
(**A**) Classification of the identified metabolites in the five analyzed legumes. The color of the dots represents the transformed P-value of the hypergeometric test, and the size represents the number of differential metabolites, and the larger the size, the greater the number of differential metabolites within the chemical class. (**B**) Top 15 different enriched KEGG pathways, the horizontal coordinate indicates the ratio of the differential metabolite numbers in the corresponding pathway to the total identified metabolite numbers in this pathway, and the larger the ratio value, the greater the enrichment of this pathway, and the vertical coordinate indicates the name of the pathway. The color intensity reflects the statistical significance of the identified pathways, the darker the color, the more affected the pathway.

The Kyoto Encyclopedia of Genes and Genomes (KEGG) database was used to link the identified metabolites (Table S1) to their metabolic pathways. Metabolite set enrichment analysis (MSEA) was utilized to classify the chemical groups of all identified compounds and highlight the most enriched metabolic pathway in the analyzed legume samples (Fig. 5). The enrichment bubble diagram (Fig. 5A) represents the chemical classifications of the enriched metabolite sets (top 25). As can be observed, among the top 25 chemical classes, the chemical groups with a higher enrichment ratio were cholines, oligosaccharides, fatty acid conjugates, flavonoids, glycerophosphoglycerols, prenol lipids, benzamides, octadecanoids, isoprenoids, and fatty acyl glycosides. The metabolic pathways of the metabolites were also analyzed according to the KEGG database, which reflected the most significant biochemical metabolic pathways involved by the identified metabolites in the five legumes. The identified metabolites covered a total of 29 pathways or metabolisms (Fig. S4, Table S2) and the top 15 enriched pathway terms are shown in the KEGG enrichment bar chart by calculating the -log(P-value) of each pathway, including glycerophospholipid metabolism, glycine, serine and threonine metabolism, unsaturated fatty acids biosynthesis, linoleic acid metabolism, ether lipid metabolism, valine, leucine and isoleucine biosynthesis, flavonoid biosynthesis, anthocyanin biosynthesis, and galactose metabolism (Fig. 5B). However, most metabolic reactions involved multiple metabolites, and the variation of these metabolites amounts among the five legume samples was inconsistent. Therefore, it cannot be simply said that the expression of some metabolic pathways was increased or decreased in a certain legume species. Previous studies reported that the major metabolic pathways in legumes included flavone and flavonol biosynthesis, aminoacyl-tRNA biosynthesis, isoquinoline alkaloid biosynthesis, the biosynthesis of amino acids, and isoflavonoid biosynthesis^82,83^ which is in accordance with our results (Table S2). Interestingly, many of the enriched metabolic pathways in the five legumes were associated with the biosynthesis of plant secondary metabolites, such as sterols, saponins, alkaloids, isoprenoids, and flavonoids. Secondary metabolites in plants are non-essential small molecular organic molecules generated by secondary metabolism and often have bioactivity. As a result, metabolic pathway analysis is beneficial for investigating complicated biological processes that occur throughout the metabolite accumulation process in plants^84^. Indeed, the characterized chemical classes in the analyzed legumes such as flavonoids, cinnamic acids, benzoic acids, alkaloids, and sterols have been proven to exhibit strong antioxidant and anti-cholinesterase activities^85–87^. In this context, potential metabolites identified in metabolic pathways might serve as therapeutic targets and contribute to the development of broad-spectrum drugs. It can aid in the development of testable predictions, the understanding of drug action mechanisms, and the increase of research productivity towards novel drug discovery^88^.

### Correlation between metabolites and anti-cholinesterase activity

**Figure 6:**
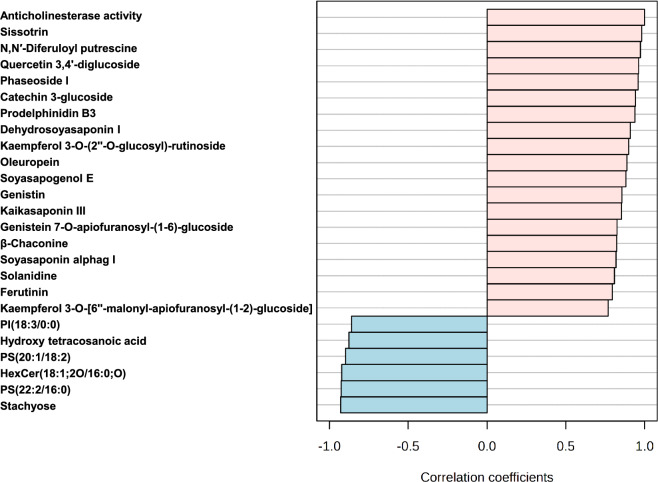
Top 25 metabolites correlated by Pearson’s correlation coefficients with the anti-cholinesterase activity of the five legumes (*p* < 0.05, *n* = 3).

The association between the identified metabolites and anti-cholinesterase activity of the tested legume extracts was established using Pearson’s correlation analysis. As can be observed in Fig. 6, certain relationships between the abundance of the identified metabolites in the five extracts and the bioactivity with Pearson’s correlation coefficient (r) r > ± 0.5 at *p* < 0.05 and false discovery rate (FDR) < 0.01. Indeed, 18 metabolites were positively correlated to the anti-cholinesterase activity of the extracts, including, 3 isoflavonoids (sissotrin, genistin, and genistein 7-O-apiofuranosyl-(1–6)-glucoside), 4 flavonoids (quercetin 3,4'-diglucoside, catechin 3-glucoside, kaempferol 3-O-(2''-O-glucosyl)-rutinoside, kaempferol 3-O-[6''-malonyl-apiofuranosyl-(1–2)-glucoside]), 5 saponins (phaseoside I, dehydro-soyasaponin I, soyasapogenol E, kaikasaponin III, soyasaponin alphag I), 2 alkaloids (β-chaconine and solanidine), and 2 phenolic acid derivative (N, N′-diferuloyl putrescine and ferutinin), as well as, the seco-iridoid oleuropein and the procyanidin, prodelphinidin B3. In contrast, only 6 metabolites exhibited a negative correlation with the activity and were mainly fatty acids and lipids. To further validate these findings a docking study was performed to explore the potential binding modes and the intermolecular interactions of the detected biomarkers with AChE and BuChE active sites.

### Docking study

#### Two molecular docking studies

The 18 positively correlated metabolites from the five seeds obtained by Pearson’s correlation analysis were docked against both AChE and BuChE targets to clarify their anti-Alzheimer effects at the molecular levels **(**Table S3**)**. The docked co-crystals of AChE and BuChE achieved binding scores of −9.51 and −8.89 kcal/mol, respectively. Based on the binding scores and binding modes; beta-chaconine, dehydro-soyasaponin I, kaempferol-3-*O*-(2''-*O*-glucosyl)-rutinoside, kaikasaponin III, *N*, *N'*-diferuloyl putrescine, and oleuropein members were found to be the most promising, Table 2. β-Chaconine described binding scores of −7.86 and −10.32 kcal/mol against the AChE and BuChE targets, respectively. It bound Trp286 (H-bond and H-pi bond), Ser293 (two H-bonds), and Phe295 (one H-bond) of AChE. Also, it bound Tyr332 (H-pi), Glu197 (two H-bonds), Gly116 (H-bond), and His438 (H-bond) of BuChE. Dehydro-soyasaponin I binding scores were − 6.37 and − 11.41 kcal/mol towards the AChE and BuChE receptors, respectively. It formed three H-bonds with Ser293 and one H-bond with Asp74 of the AChE; besides, it formed one H-bond with Asn289 and one H-bond with Thr332 of the BuChE. Moreover, kaempferol-3-*O*-(2''-*O*-glucosyl)-rutinoside achieved binding scores of -8.33 and -10.30 kcal/mol within the binding pockets of AChE and BuChE, respectively. It showed the formation of two H-pi interactions with Trp286, two H-bonds with Ser293, and one H-bond with Phe295 of AChE. However, it formed two H-bonds with Asp70 and one H-bond with Asn289 of BuChE. Furthermore, kaikasaponin III showed binding scores of−5.77 and − 11.36 kcal/mol against the binding sites of AChE and BuChE, respectively. Kaikasaponin III represented two H-bonds with Ser293 and one H-bond with Glu292 of the AChE receptor: besides, one H-bond with Asn289 and one H-bond with Tyr332 of the BuChE. *N*, *N'*-diferuloyl putrescine on the other side was found to interact with the binding sites of AChE and BuChE achieving scores of −9.57 and −8.41 kcal/mol, respectively. It bound both Trp286 and Glu202 amino acids of AChE with pi-H and H-bond, respectively. Also, it bound both Asp70 and Gly116 amino acids of BuChE with H-bond and pi-H interaction, respectively. Finally, oleuropein formed one H-bond with Asp74 and one pi–pi interaction with Tyr341 of the AChE receptor and showed a binding score of −9.28 kcal/mol. Besides, it bound Ser287 (H-bond), Gly 116 (H-bond), and Tyr332 (H-pi bond) of BuChE and described a binding score of −9.25 kcal/mol. Briefly, the aforementioned findings suggest the very promising inhibitory activities of the identified metabolites towards the AChE and BuChE targets.

**Table 2:** 3D pocket interactions and positioning for beta-chaconine, dehydro-soyasaponin I, kaempferol-3-*O*-(2''-*O*-glucosyl)-rutinoside, kaikasaponin III, *N*, *N'*-diferuloyl putrescine, and oleuropein towards the AChE and BuChE targets.

Comp	Receptor	3D interactions	3D positioning
Beta-Chaconine	AChE		
	BuChE		
Dehydro-Soyasaponin I	AChE		
	BuChE		
Kaempferol-3-O-(2''-O-glucosyl)-rutinoside	AChE		
	BuChE		
Kaikasaponin III	AChE		
	BuChE		
*N*,*N'*-Diferuloyl putrescine	AChE		
	BuChE		
Oleuropein	AChE		
	BuChE		

## Conclusions

The chemometric study of five commonly used legumes (FB, BP, KB, RL, and CP) in addition to their antioxidant and anti-cholinesterase activities justified by the computational analysis was the main core of our study. Their comprehensive profiles were studied for the first time showing the top 25 differential metabolites. The metabolic pathway enrichment analysis based on KEGG database revealed that many of the enriched metabolic pathways in the five investigated legumes were associated with the biosynthesis of plant secondary metabolites, like sterols, saponins, alkaloids, isoprenoids, and flavonoids. The biological activity of the samples reflected the potential of legumes against the key enzymes linked to Alzheimer’s disease. Where the daily consumption of CP will be recommended for the elderly to protect their nerves. Eighteen metabolites were positively correlated with the anti-cholinesterase activities. This was justified by their promising binding scores upon docking to the active sites of AChE and BuChE. The current study highly recommends further in vivo and clinical studies of the legume seeds, either alone or in combinations as possible anticholinergic agents, to allow for the formulation and rational planning of new natural anti-Alzheimer’s drugs.

### Supplementary Information

Supplementary Information.

## Data Availability

The authors declare that the data supporting the findings of this study are available within the paper and its Supplementary Information files.

## References

[1] Noori, T., Dehpour, A. R., Sureda, A., Sobarzo-Sanchez, E. & Shirooie, S. Role of natural products for the treatment of Alzheimer’s disease. *Eur. J. Pharmacol.* **898**, 173974 (2021). 33652057 10.1016/j.ejphar.2021.173974

[2] Fancellu, G. *et al.* Novel tacrine–benzofuran hybrids as potential multi-target drug candidates for the treatment of Alzheimer’s Disease. *J. Enzym. Inhib. Med. Chem.* **35**, 211–226 (2020). 10.1080/14756366.2019.1689237 PMC7567501 31760822

[3] Gaudreault, R. & Mousseau, N. Mitigating Alzheimer’s disease with natural polyphenols: A review. *Curr. Alzheimer Res.* **16**, 529–543 (2019). 30873922 10.2174/1567205016666190315093520

[4] Ayaz, M., Ullah, F., Sadiq, A., Kim, M. O. & Ali, T. Editorial: Natural products-based drugs: Potential therapeutics against Alzheimer’s disease and other neurological disorders. *Front. Pharmacol.*10.3389/fphar.2019.01417 (2019). 31849668 10.3389/fphar.2019.01417 PMC6889855

[5] Caprioli, G. *et al.* Lipid nutritional value of legumes: Evaluation of different extraction methods and determination of fatty acid composition. *Food Chem.* **192**, 965–971 (2016). 26304436 10.1016/j.foodchem.2015.07.102

[6] Curiel, J. A. *et al.* Exploitation of the nutritional and functional characteristics of traditional Italian legumes: The potential of sourdough fermentation. *Int. J. Food Microbiol.* **196**, 51–61 (2015). 25522057 10.1016/j.ijfoodmicro.2014.11.032

[7] Margier, M. *et al.* Nutritional composition and bioactive content of legumes: Characterization of pulses frequently consumed in France and effect of the cooking method. *Nutrients* **10**, 1668 (2018). 30400385 10.3390/nu10111668 PMC6266829

[8] Ha, T. J. *et al.* Rapid characterisation and comparison of saponin profiles in the seeds of Korean Leguminous species using ultra performance liquid chromatography with photodiode array detector and electrospray ionisation/mass spectrometry (UPLC–PDA–ESI/MS) analysis. *Food Chem.* **146**, 270–277 (2014). 24176342 10.1016/j.foodchem.2013.09.051

[9] Castañeda, F. N., Vidal, R. B. P. & Aspromonte, J. Untargeted chromatographic methods coupled with chemometric strategies for the analysis of food and related samples. *TrAC Trends Anal. Chem.*10.1016/j.trac.2024.117650 (2024). 10.1016/j.trac.2024.117650

[10] Ibrahim, R. M. *et al.* LC/MS-based metabolomics reveals chemical variations of two broccoli varieties in relation to their anticholinesterase activity: In vitro and in silico studies. *Plant Foods Hum. Nutr.*10.1007/s11130-024-01161-2 (2024). 38607508 10.1007/s11130-024-01161-2 PMC11178554

[11] Maldini, M. *et al.* Untargeted metabolomics reveals predominant alterations in lipid metabolism following light exposure in broccoli sprouts. *Int. J. Mol. Sci.* **16**, 13678–13691 (2015). 26084047 10.3390/ijms160613678 PMC4490517

[12] Castro-Puyana, M. & Herrero, M. Metabolomics approaches based on mass spectrometry for food safety, quality and traceability. *TrAC Trends Anal. Chem.* **52**, 74–87 (2013). 10.1016/j.trac.2013.05.016

[13] Cubero-Leon, E., Peñalver, R. & Maquet, A. Review on metabolomics for food authentication. *Food Res. Int.* **60**, 95–107 (2014). 10.1016/j.foodres.2013.11.041

[14] Lučić, D. *et al.* Antioxidant and antiproliferative activities of kale (*Brassica**oleracea* L. *Var**acephala* DC.) and wild cabbage (*Brassica**incana* Ten.) polyphenolic extracts. *Molecules* **28**, 1840 (2023). 36838827 10.3390/molecules28041840 PMC9958672

[15] Li, P. *et al.* Widely targeted metabolomics analysis of soybean and chickpea and their different advantages and new functional compounds for diabetes. *Molecules* **27**, 5297 (2022). 36014535 10.3390/molecules27165297 PMC9413387

[16] Konar, N., Poyrazoğlu, E. S., Demir, K. & Artik, N. Determination of conjugated and free isoflavones in some legumes by LC–MS/MS. *J. Food Compos. Anal.* **25**, 173–178 (2012). 10.1016/j.jfca.2011.11.004

[17] Pal, L. *et al.* Biochemical analysis of anthocyanin and proanthocyanidin and their regulation in determining chickpea flower and seed coat colour. *J. Exp. Bot.* **74**, 130–148 (2023). 36205079 10.1093/jxb/erac392

[18] Sagratini, G. *et al.* Determination of soyasaponins I and βg in raw and cooked legumes by solid phase extraction (SPE) coupled to liquid chromatography (LC)–mass spectrometry (MS) and assessment of their bioaccessibility by an in vitro digestion model. *J. Agric. Food Chem.* **61**, 1702–1709 (2013). 23305351 10.1021/jf304136g

[19] Kwon, S.-J. *et al.* Phytochemical compounds and antioxidant activity in the grain of selected faba bean (*Vicia**faba*) genotypes. *Plant Breed. Biotechnol.* **6**, 65–73 (2018). 10.9787/PBB.2018.6.1.65

[20] Sedláková, V., Zeljković, S. Ć, Štefelová, N., Smýkal, P. & Hanáček, P. Phenylpropanoid content of chickpea seed coats in relation to seed dormancy. *Plants* **12**, 2687 (2023). 37514301 10.3390/plants12142687 PMC10384132

[21] Sagratini, G. *et al.* Quantification of soyasaponins I and βg in Italian lentil seeds by solid-phase extraction (SPE) and high-performance liquid chromatography− mass spectrometry (HPLC-MS). *J. Agric. Food Chem.* **57**, 11226–11233 (2009). 19950999 10.1021/jf901707z

[22] Llorach, R. *et al.* Comparative metabolite fingerprinting of legumes using LC-MS-based untargeted metabolomics. *Food Res. Int.* **126**, 108666. 10.1016/j.foodres.2019.108666 (2019). 31732019 10.1016/j.foodres.2019.108666

[23] El-Shiekh, R. A., Shalabi, A. A., Al-Hawshabi, O. S., Salkini, M. A. & Abdel-Sattar, E. Anticholinesterase and anti-inflammatory constituents from *Caralluma awdeliana,* a medicinal plant from Yemen. *Steroids* **193**, 109198 (2023). 36780968 10.1016/j.steroids.2023.109198

[24] El-Shiekh, R. A., Kassem, H. A., Khaleel, A. E. & Abd El-Mageed, M. M. Anticholinesterases activity of *Murraya**koenigii* (L.) Spreng. and *Murraya**paniculata* (L.) Jacq. essential oils with GC/MS analysis and molecular docking. *Nat. Prod. Res.* **38**, 2155–2159 (2023). 37516925 10.1080/14786419.2023.2241150

[25] Hamed, A. A. *et al.* Cholinesterase inhibitors from an endophytic fungus *Aspergillus**niveus* Fv-er401: Metabolomics, isolation and molecular docking. *Molecules* **28**, 2559 (2023). 36985531 10.3390/molecules28062559 PMC10052609

[26] Hussein, M. E. *et al.* Anticholinesterase activity of budmunchiamine alkaloids revealed by comparative chemical profiling of two Albizia spp., molecular docking and dynamic studies. *Plants* **11**, 3286 (2022). 36501324 10.3390/plants11233286 PMC9738009

[27] El-Shiekh, R. A., Ashour, R. M., Abd El-Haleim, E. A., Ahmed, K. A. & Abdel-Sattar, E. Hibiscus sabdariffa L.: A potent natural neuroprotective agent for the prevention of streptozotocin-induced Alzheimer’s disease in mice. *Biomed. Pharmacother.* **128**, 110303 (2020). 32480228 10.1016/j.biopha.2020.110303

[28] Mahdy, N. E. *et al.* Hepatoprotective mechanisms of chemically characterized Aloe striata gel with and without loading on nanoparticles, involving ERK-JNK signaling pathway. *S. Afr. J. Bot.* **170**, 163–171 (2024). 10.1016/j.sajb.2024.05.024

[29] Khuda, F. *et al.* Biosynthesized silver nanoparticles using *Alnus**nitida* leaf extract as a potential antioxidant and anticancer agent. *ACS Omega* **8**, 30221–30230 (2023). 37636925 10.1021/acsomega.3c02928 PMC10448672

[30] Rahman, M. M., Islam, M. B., Biswas, M. & Khurshid Alam, A. In vitro antioxidant and free radical scavenging activity of different parts of *Tabebuia**pallida* growing in Bangladesh. *BMC Res. Notes* **8**, 1–9 (2015). 26518275 10.1186/s13104-015-1618-6 PMC4627625

[31] Srour, A. M. *et al.* Design, synthesis and molecular docking simulation of oxindole-based derivatives with dual VEGFR-2 and cholinesterase inhibitory activities. *J. Mol. Struct.* **1271**, 134130 (2022). 10.1016/j.molstruc.2022.134130

[32] Fawazy, N. G. *et al.* Development of spiro-3-indolin-2-one containing compounds of antiproliferative and anti-SARS-CoV-2 properties. *Sci. Rep.* **12**, 1–21 (2022). 35974029 10.1038/s41598-022-17883-9 PMC9380671

[33] Hussein, H. M. *et al.* New insight into the anticholinesterase potential of *Cordia**dichotoma* G. Forst. and *Cordia**sebestena* L. leaves, phenolic characterization of their active extracts by HPLC-DAD and molecular modeling. *Egypt. J. Chem.* **67**, 225–237 (2024).

[34] Hussein, M. E. *et al.* Identification of antibacterial metabolites from endophytic fungus *Aspergillus**fumigatus*, isolated from *Albizia**lucidior* leaves (Fabaceae), utilizing metabolomic and molecular docking techniques. *Molecules* **27**, 1117 (2022). 35164382 10.3390/molecules27031117 PMC8839868

[35] Ibrahim, R. M., Elmasry, G. F., Refaey, R. H. & El-Shiekh, R. A. *Lepidium**meyenii* (maca) roots: UPLC-HRMS, molecular docking, and molecular dynamics. *ACS Omega* **7**, 17339–17357 (2022). 35647470 10.1021/acsomega.2c01342 PMC9134390

[36] Inc, C. Molecular operating environment (MOE). *Chemical Computing Group Inc* **1010** (2016).

[37] Elshal, M., Eid, N., El-Sayed, I., El-Sayed, W. & Al-Karmalawy, A. A. Concanavalin-A shows synergistic cytotoxicity with Tamoxifen via inducing apoptosis in estrogen receptor-positive breast cancer: In vitro and molecular docking studies. *Pharm. Sci.* **28**, 76–85 (2021).

[38] Khattab, M. & Al-Karmalawy, A. A. Computational repurposing of benzimidazole anthelmintic drugs as potential colchicine binding site inhibitors. *Future Med. Chem.* **13**, 1623–1638. 10.4155/fmc-2020-0273 (2021). 34505541 10.4155/fmc-2020-0273

[39] Taher, R. F. *et al.* Two new flavonoids and anticancer activity of *Hymenosporum**flavum*: In vitro and molecular docking studies. *J. Herbmed. Pharmacol.* **10**, 443–458. 10.34172/jhp.2021.52 (2021). 10.34172/jhp.2021.52

[40] Elmaaty, A. A. *et al.* Anticoagulants as potential SARS-CoV-2 Mpro inhibitors for COVID-19 patients: In vitro, molecular docking, molecular dynamics, DFT, and SAR studies. *Int. J. Mol. Sci.* **23**, 12235 (2022). 36293094 10.3390/ijms232012235 PMC9603561

[41] Elagawany, M. *et al.* Ligand-based design, synthesis, computational insights, and in vitro studies of novel N-(5-Nitrothiazol-2-yl)-carboxamido derivatives as potent inhibitors of SARS-CoV-2 main protease. *J. Enzyme Inhib. Med. Chem.* **37**, 2112–2132. 10.1080/14756366.2022.2105322 (2022). 35912578 10.1080/14756366.2022.2105322 PMC9344964

[42] Al-Karmalawy, A. A. *et al.* Design and statistical optimisation of emulsomal nanoparticles for improved anti-SARS-CoV-2 activity of N-(5-nitrothiazol-2-yl)-carboxamido candidates: In vitro and in silico studies. *J. Enzyme Inhib. Med. Chem.* **38**, 2202357. 10.1080/14756366.2023.2202357 (2023). 37092260 10.1080/14756366.2023.2202357 PMC10128464

[43] Wibowo, A. *et al.* Malaysianol A, a new trimer resveratrol oligomer from the stem bark of *Dryobalanops**aromatica*. *Fitoterapia* **82**, 676–681 (2011). 21338657 10.1016/j.fitote.2011.02.006

[44] Amarowicz, R. & Pegg, R. B. Legumes as a source of natural antioxidants. *Eur. J. Lipid Sci. Technol.* **110**, 865–878 (2008). 10.1002/ejlt.200800114

[45] Lin, C.-C., Wu, S.-J., Wang, J.-S., Yang, J.-J. & Chang, C.-H. Evaluation of the antioxidant activity of legumes. *Pharm. Biol.* **39**, 300–304 (2001). 10.1076/phbi.39.4.300.5919

[46] Siddhuraju, P. & Becker, K. The antioxidant and free radical scavenging activities of processed cowpea (*Vigna**unguiculata* (L.) Walp.) seed extracts. *Food Chem.* **101**, 10–19 (2007). 10.1016/j.foodchem.2006.01.004

[47] Orita, A., Musou-Yahada, A., Shoji, T., Oki, T. & Ohta, H. Comparison of anthocyanins, proanthocyanidin oligomers and antioxidant capacity between cowpea and grain legumes with colored seed coat. *Food Sci. Technol. Res.* **25**, 287–294 (2019). 10.3136/fstr.25.287

[48] Kan, L. *et al.* Comparative study on the chemical composition, anthocyanins, tocopherols and carotenoids of selected legumes. *Food Chem.* **260**, 317–326 (2018). 29699675 10.1016/j.foodchem.2018.03.148

[49] Mazur, W. M., Duke, J. A., Wähälä, K., Rasku, S. & Adlercreutz, H. Isoflavonoids and lignans in legumes: Nutritional and health aspects in humans. *J. Nutr. Biochem.* **9**, 193–200 (1998). 10.1016/S0955-2863(97)00184-8

[50] Zou, Y., Chang, S. K., Gu, Y. & Qian, S. Y. Antioxidant activity and phenolic compositions of lentil (*Lens**culinaris* var. Morton) extract and its fractions. *J. Agric. Food Chem.* **59**, 2268–2276 (2011). 21332205 10.1021/jf104640k PMC3063125

[51] Konar, N. Non-isoflavone phytoestrogenic compound contents of various legumes. *Eur. Food Res. Technol.* **236**, 523–530 (2013). 10.1007/s00217-013-1914-0

[52] Schymanski, E. L. *et al.* Strategies to characterize polar organic contamination in wastewater: Exploring the capability of high resolution mass spectrometry. *Environ. Sci. Water Res.* **48**(3), 1811–1818 (2014). 10.1021/es4044374 24417318

[53] Sumner, L. W. *et al.* Proposed minimum reporting standards for chemical analysis: Chemical analysis working group (CAWG) metabolomics standards initiative (MSI). *Metabolomics* **3**, 211–221 (2007). 24039616 10.1007/s11306-007-0082-2 PMC3772505

[54] Arce, M. P. *et al.* Neuroprotective and cholinergic properties of multifunctional glutamic acid derivatives for the treatment of Alzheimer’s disease. *J. Med. Chem.* **52**, 7249–7257 (2009). 19856923 10.1021/jm900628z

[55] Lee, W. S. *et al.* The potential neuroprotective effects of extracts from oat seedlings against Alzheimer’s disease. *Nutrients* **14**, 4103 (2022). 36235754 10.3390/nu14194103 PMC9571310

[56] Takata, M. K. *et al.* Neuroprotective effect of D-psicose on 6-hydroxydopamine-induced apoptosis in rat pheochromocytoma (PC12) cells. *J. Biosci. Bioeng.* **100**, 511–516 (2005). 16384789 10.1263/jbb.100.511

[57] Putteeraj, M., Lim, W. L., Teoh, S. L. & Yahaya, M. F. Flavonoids and its neuroprotective effects on brain ischemia and neurodegenerative diseases. *Curr. Drug Targets* **19**, 1710–1720 (2018). 29577854 10.2174/1389450119666180326125252

[58] Szwajgier, D., Borowiec, K. & Pustelniak, K. The neuroprotective effects of phenolic acids: Molecular mechanism of action. *Nutrients* **9**, 477 (2017). 28489058 10.3390/nu9050477 PMC5452207

[59] Vallejo, F., Tomás-Barberán, F. & Ferreres, F. Characterisation of flavonols in broccoli (*Brassica oleracea* L. var. *italica*) by liquid chromatography–UV diode-array detection–electrospray ionisation mass spectrometry. *J. Chromatogr. A* **1054**, 181–193 (2004). 15553143 10.1016/j.chroma.2004.05.045

[60] Mekky, R. H. *et al.* Profiling of phenolic and other compounds from Egyptian cultivars of chickpea (*Cicer**arietinum* L.) and antioxidant activity: A comparative study. *RSC Adv.* **5**, 17751–17767 (2015). 10.1039/C4RA13155J

[61] Luna, L. C. *et al.**Ramorinoa**girolae* Speg (Fabaceae) seeds, an Argentinean traditional indigenous food: Nutrient composition and antioxidant activity. *J. Food Compos. Anal.* **31**, 120–128 (2013). 10.1016/j.jfca.2013.05.004

[62] Khole, S. *et al.* Bioactive constituents of germinated fenugreek seeds with strong antioxidant potential. *J. Funct. Foods* **6**, 270–279 (2014). 10.1016/j.jff.2013.10.016

[63] Talhi, O. & Silva, A. Organic synthesis of C-prenylated phenolic compounds. *Curr. Org. Chem.* **17**, 1067–1102 (2013). 10.2174/1385272811317100009

[64] Wang, C. *et al.* Iridoids: Research advances in their phytochemistry, biological activities, and pharmacokinetics. *Molecules* **25**, 287 (2020). 31936853 10.3390/molecules25020287 PMC7024201

[65] Mir, R. H. *et al.* Natural anti-inflammatory compounds as drug candidates in Alzheimer’s disease. *Curr. Med. Chem.* **28**, 4799–4825 (2021). 32744957 10.2174/0929867327666200730213215

[66] Wojtunik-Kulesza, K. *et al.* Selected natural products in neuroprotective strategies for Alzheimer’s disease—a non-systematic review. *Int. J. Mol. Sci.* **23**, 1212 (2022). 35163136 10.3390/ijms23031212 PMC8835836

[67] Liu, X. *et al.* Lignans from the root of *Paeonia**lactiflora* and their anti-β-amyloid aggregation activities. *Fitoterapia* **103**, 136–142 (2015). 25818229 10.1016/j.fitote.2015.03.011

[68] Savarese, M., De Marco, E. & Sacchi, R. Characterization of phenolic extracts from olives (Olea europaea cv. Pisciottana) by electrospray ionization mass spectrometry. *Food Chem.* **105**, 761–770 (2007). 10.1016/j.foodchem.2007.01.037

[69] Sicilia, T., Niemeyer, H. B., Honig, D. M. & Metzler, M. Identification and stereochemical characterization of lignans in flaxseed and pumpkin seeds. *J. Agric. Food Chem.* **51**, 1181–1188 (2003). 12590454 10.1021/jf0207979

[70] Kumar, G. P. & Khanum, F. Neuroprotective potential of phytochemicals. *Pharmacogn. Rev.* **6**, 81 (2012). 23055633 10.4103/0973-7847.99898 PMC3459459

[71] Bianco, G. *et al.* Determination of soyasaponins in Fagioli di Sarconi beans (*Phaseolus**vulgaris* L.) by LC-ESI-FTICR-MS and evaluation of their hypoglycemic activity. *Anal. Bioanal. Chem.* **410**, 1561–1569 (2018). 29270658 10.1007/s00216-017-0806-8

[72] Nielsen, S. D., Schmidt, J. M., Kristiansen, G. H., Dalsgaard, T. K. & Larsen, L. B. Liquid chromatography mass spectrometry quantification of α-solanine, α-chaconine, and solanidine in potato protein isolates. *Foods* **9**, 416 (2020). 32252270 10.3390/foods9040416 PMC7230682

[73] Nikolic, N. C. & Stankovic, M. Z. Solanidine hydrolytic extraction and separation from the potato (*Solanum**tuberosum* L.) vines by using solid− liquid− liquid systems. *J. Agric. Food Chem.* **51**, 1845–1849 (2003). 12643640 10.1021/jf020426s

[74] Hwang, J. P., Ha, J. H., No, G. Y., Jeong, Y. J. & Park, S. N. Cellular protective effect of novel dimeric ferulamide derivatives against UVA and 1O2 and its structural mechanism. *J. Ind. Eng. Chem.* **53**, 164–170 (2017). 10.1016/j.jiec.2017.04.021

[75] Dong, X. *et al.* Spatiotemporal distribution of phenolamides and the genetics of natural variation of hydroxycinnamoyl spermidine in rice. *Mol. Plant* **8**, 111–121 (2015). 25578276 10.1016/j.molp.2014.11.003

[76] de Pascual-Teresa, S. Molecular mechanisms involved in the cardiovascular and neuroprotective effects of anthocyanins. *Arch. Biochem. Biophys.* **559**, 68–74 (2014). 24791600 10.1016/j.abb.2014.04.012

[77] Escobar-Avello, D. *et al.* Phenolic profile of grape canes: Novel compounds identified by LC-ESI-LTQ-Orbitrap-MS. *Molecules* **24**, 3763 (2019). 31635434 10.3390/molecules24203763 PMC6832258

[78] Razgonova, M. *et al.* LC-MS/MS screening of phenolic compounds in wild and cultivated grapes *Vitis**amurensis* Rupr.. *Molecules* **26**, 3650 (2021). 34203808 10.3390/molecules26123650 PMC8232594

[79] Hashimoto, M. & Hossain, S. Neuroprotective and ameliorative actions of polyunsaturated fatty acids against neuronal diseases: Beneficial effect of docosahexaenoic acid on cognitive decline in Alzheimer’s disease. *J. Pharm. Sci.* **116**, 150–162 (2011). 10.1254/jphs.10R33FM 21606627

[80] Otify, A. M. *et al.* Unveiling metabolome heterogeneity and new chemicals in 7 tomato varieties via multiplex approach of UHPLC-MS/MS, GC–MS, and UV–Vis in relation to antioxidant effects as analyzed using molecular networking and chemometrics. *Food Chem.* **417**, 135866. 10.1016/j.foodchem.2023.135866 (2023). 36913868 10.1016/j.foodchem.2023.135866

[81] Zhang, J. *et al.* Characterization of phospholipids from Pacific saury (*Cololabis**saira*) viscera and their neuroprotective activity. *Food Biosci.* **24**, 120–126 (2018). 10.1016/j.fbio.2018.06.002

[82] Shi, S.-H. *et al.* Comparative metabolomic profiling reveals key secondary metabolites associated with high quality and nutritional value in broad bean (*Vicia**faba* L.). *Molecules* **27**, 8995 (2022). 36558128 10.3390/molecules27248995 PMC9787534

[83] Khan, M. A. *et al.* Transcriptome profiling of faba bean (*Vicia**faba* L.) drought-tolerant variety hassawi-2 under drought stress using RNA sequencing. *Electron. J. Biotechnol.* **39**, 15–29 (2019). 10.1016/j.ejbt.2019.02.004

[84] Al-Nemi, R., Makki, A. A., Sawalha, K., Hajjar, D. & Jaremko, M. Untargeted metabolomic profiling and antioxidant capacities of different solvent crude extracts of *Ephedra**foeminea*. *Metabolites* **12**, 451 (2022). 35629955 10.3390/metabo12050451 PMC9146585

[85] Orhan, I., Kartal, M., Tosun, F. & Şener, B. Screening of various phenolic acids and flavonoid derivatives for their anticholinesterase potential. *Zeitschrift für Naturforschung C* **62**, 829–832 (2007). 10.1515/znc-2007-11-1210 18274286

[86] Ogunsuyi, O. B., Ademiluyi, A. O. & Oboh, G. Solanum leaves extracts exhibit antioxidant properties and inhibit monoamine oxidase and acetylcholinesterase activities (in vitro) in *Drosophila**melanogaster*. *J. Basic Clin. Physiol. Pharmacol.*10.1515/jbcpp-2019-0256 (2020). 32267245 10.1515/jbcpp-2019-0256

[87] Bari, W. U. *et al.* Anticholinesterase, antioxidant potentials, and molecular docking studies of isolated bioactive compounds from *Grewia**optiva*. *Int. J. Food Prop.* **22**, 1386–1396 (2019). 10.1080/10942912.2019.1650763

[88] Wang, X., Yang, B., Zhang, A., Sun, H. & Yan, G. Potential drug targets on insomnia and intervention effects of Jujuboside A through metabolic pathway analysis as revealed by UPLC/ESI-SYNAPT-HDMS coupled with pattern recognition approach. *J. Proteom.* **75**, 1411–1427 (2012). 10.1016/j.jprot.2011.11.011 22134358

