# Supplementary material for: Metabolites profiling, in-vitro and molecular docking studies of five legume seeds for Alzheimer’s disease
Source: Sci Rep. 2024 Aug 23;14:19637. doi: 10.1038/s41598-024-68743-7 (PMC11344142; doi:10.1038/s41598-024-68743-7)
Supplement: Supplementary file 1 — Supplementary Information. [file 41598_2024_68743_MOESM1_ESM.pdf]

Table S1. Annotated metabolites in the Five commonly used legumes (Fabaceae).

**Identification**	**Retention time (min)**	***m/z***	**Mode**	**Chemical class**	**Exact Mass**	**MF**	**FB**	**Bp**	**KB**	**RL**	**CP**	**Mass fragments**	**Ref.**	**Reported***
β-Chaconine	4.23	706.4173	P	Alkaloid	705.445199	$C_{39}H_{63}NO_{10}$	+	+	+	+	+	559, 397	[1]	-
Solanidine	4.48	398.2414	P	Alkaloid	397.334464	$C_{27}H_{43}NO$	-	+	-	+	+	381, 204, 150	[1]	-
Thermospermine	5.37	203.1791	P	Amine derivative	202.215746	$C_{10}H_{26}N_4$	+	+	+	+	+	185	Sirus	-
Choline	0.39	104.1068	P	Amine derivative	104.107539	$C_5H_{14}NO$	+	+	+	+	+	-	Sirus	[2]
Tyramine	0.42	138.0547	P	Amine derivative	137.084064	$C_8H_{11}NO$	-	-	-	+	-	121	HMDB	[2]
Isovaline	0.42	118.086	P	Amino acid	117.078979	$C_5H_{11}NO_2$	-	-	-	+	+	119	HMDB	[2]
Pipecolic acid	0.43	130.0862	P	Amino acid	129.078979	$C_6H_{11}NO_2$	+	+	+	+	+	129	HMDB	[2]
Glu-Tyr	0.56	311.1238	P	Amino acid	310.116488	$C_{14}H_{18}N_2O_6$	+	+	+	+	+	137	HMDB	[2]
Glu-Leu	0.71	261.1444	P	Amino acid	260.137223	$C_{11}H_{20}N_2O_5$	-	-	-	+	-	131	HMDB	[2]
Isoleucine	0.71	132.1016	P	Amino acid	131.094629	$C_6H_{13}NO_2$	+	-	-	+	+	114	HMDB	[2]
Glu-Phe	0.98	295.1287	P	Amino acid	294.121573	$C_{14}H_{18}N_2O_5$	+	-	-	+	-	121	HMDB	[2]
Pelargonidin 3-*O*-glucoside	2.25	433.1129	P	Proanthocyanidin	432.105649	$C_{21}H_{20}O_{10}$	-	-	-	+	-	271	[3]	[3]
Prodelphinidin B3	4.69	595.3119	P	Proanthocyanidin	594.137345	$C_{30}H_{26}O_{13}$	-	+	+	-	-	591, 565, 467, 425, 423, 407, 305	[4]	[4]
Procyanidin dimer B7	4.87	579.2925	P	Proanthocyanidin	578.14243	$C_{30}H_{26}O_{12}$	-	-	-	-	+	451, 425, 407, 289	[5]	[5]
Trihydroxy methoxy benzophenone	0.71	259.1293	N	Benzophenones	260.068475	$C_{14}H_{12}O_5$	-	+	+	-	+	245, 229, 213, 211	Sirus	-

Identification	Retention time (min)	*m/z*	Mode	Chemical class	Exact Mass	MF	FB	Bp	KB	RL	CP	Mass fragments	Ref.	Reported
Malyngic Acid	3.64	327.2164	N	Fatty acid	328.224975	$C_{18}H_{32}O_5$	-	+	+	-	-	283, 143	Sirus	[6]
10-oxo-nonadecanoic acid	4.93	313.2735	P	Fatty acid	312.266445	$C_{19}H_{36}O_3$	+	+	+	+	-	295	Sirus	Sirus
Hydroxy tetracosanoic acid	5.58	383.2214	N	Fatty acid	384.360345	$C_{24}H_{48}O_3$	-	-	+	-	+	367, 143, 125	Sirus	Sirus
16-hydroxy palmitic acid	5.68	271.2267	N	Fatty acid	272.235145	$C_{16}H_{32}O_3$	-	-	-	+	-	255, 125	Sirus	Sirus
Linoleic acid	5.86	277.2164	N	Fatty acid	278.22458	$C_{18}H_{30}O_2$	-	-	+	-	-	141, 125	Sirus	Sirus
Linoleic Acid	6.07	279.232	N	Fatty acid	280.24023	$C_{18}H_{32}O_2$	-	+	+	-	-	143	Sirus	Sirus
Octadecadien-1-ol	6.29	267.2682	P	Fatty alcohol	266.260965	$C_{18}H_{34}O$	-	-	-	+	-	251	Sirus	Sirus
β-Acetoxy olean-en-oic acid	6.52	497.362	N	Triterpene	498.37091	$C_{32}H_{50}O_4$	-	-	-	+	-	437	Sirus	Sirus
Coumaroyl caffeoyl palmitic acid derivative	4.26	565.266	N	Fatty acid derivative	566.324355	$C_{34}H_{46}O_7$	-	+	-	-	-	309	Sirus	Sirus
Tuberonic acid glucoside I	1.35	387.1643	N	Fatty acid derivative	388.173335	$C_{18}H_{28}O_9$	-	-	+	-	-	369, 225, 207, 163	[7]	[7]
Tuberonic acid glucoside II	1.64	387.1645	N	Fatty acid derivative	388.173335	$C_{18}H_{28}O_9$	-	+	+	-	+	369, 225, 207, 163	[7]	[7]
N, N'-Diferuloyl putrescine	4.21	441.3725	P	Phenolic acid derivative	440.194738	$C_{24}H_{28}N_2O_6$	-	+	+	-	-	265, 177, 145, 117	Sirus	-
Dihydro-(epi)catechin diglucoside	0.46	615.1519	N	Flavonoids	616.20034	$C_{27}H_{36}O_{16}$	-	+	+	-	-	453, 301, 291, 247	[4]	[4]

Identification	Retention time (min)	*m/z*	Error (ppm)	Mode	Chemical class	Exact Mass	MF	FB	Bp	KB	RL	CP	Mass fragments	Ref.	Reported
Kaempferol-3-O-[6"-malonyl-apiofuranosyl-(1→2)-glucoside] glucoside	0.50	827.265	0.0035	N	Flavonoids	828.196045	$C_{35}H_{40}O_{23}$	+	+	+	+	+	665, 285, 178, 151	[4]	[5]
Kaempferol-3-O-[6"-malonyl-apiofuranosyl-(1→2)-glucoside]	0.55	665.2131	-0.0015	N	Flavonoids	666.14322	$C_{29}H_{30}O_{18}$	+	-	-	+	-	503, 285, 178, 151	[4]	[5]
Catechin 3-O-glucoside	0.72	451.1239	-0.007	N	Flavonoids	452.131865	$C_{21}H_{24}O_{11}$	-	+	+	-	-	289, 271	[4]	[4]
Kaempferol-3-[galactosyl-(1->6)-glucoside] 7-[rhamnosyl-(1->3)-rhamnoside]	2.22	901.2592	-0.0031	N	Flavonoids	902.26921	$C_{39}H_{50}O_{24}$	-	+	+	-	-	755, 593, 285, 178, 151	[4]	[5]
Kaempferol-3-O-(2"-O- glucosyl)-rutinoside	2.28	755.2009	-0.0029	N	Flavonoids	756.211299	$C_{33}H_{40}O_{20}$	-	-	+	-	-	593, 285, 178, 151	[4]	[5]
Quercetin 3,7-diglucoside/ Quercetin 3,4'-diglucoside	2.50	625.1384	-0.0026	N	Flavonoids	626.148305	$C_{27}H_{30}O_{17}$	-	+	+	-	-	463, 301, 151	[4]	[4]
Stachyose	0.37	665.2132	-0.0015	N	Sugar	666.221865	$C_{24}H_{42}O_{21}$	-	-	+	-	-	383, 341	[4]	[4]
Galactosyl ciceritol	0.37	679.2283	-0.0019	N	Sugar	680.237515	$C_{25}H_{44}O_{21}$	+	-	-	-	+	611, 383, 341	[4]	[4]
Raffinose	0.37	503.1607	-0.0010	N	Sugar	504.16904	$C_{18}H_{32}O_{16}$	+	+	+	-	+	341, 179, 161	[4]	[4]
Sucrose	0.38	341.1082	-0.0007	N	Sugar	342.116215	$C_{12}H_{22}O_{11}$	-	-	-	-	-	161	[4]	[4]
Dehydro-ciceritol	0.40	515.2083	0.0014	N	Sugar	516.16904	$C_{19}H_{32}O_{16}$	+	+	+	+	+	335	[4]	[4]
Hydroxy-ferutinin	4.56	373.2025	-0.0005	N	Phenolic derivatives	374.209325	$C_{22}H_{30}O_5$	+	+	+	+	+	357	Sirus	-

Identification	Retention time (min)	*m/z*	Error (ppm)	Mode	Chemical class	Exact Mass	MF	FB	Bp	KB	RL	CP	Mass fragments	Ref.	Reported
Ferutinine	5.37	357.2061	-0.0010	N	Phenolic acid derivative	358.21441	$C_{22}H_{30}O_4$	-	+	+	-	+	341, 241	Sirus	-
Biochanin A 7-*O*-glucoside/Sissotrin	3.65	447.1286	-0.0013	P	Isoflavonoid	446.1213	$C_{22}H_{22}O_{10}$	-	+	-	-	+	283, 267, 133	[7]	[7]
6"-O-Malonyl Genistin	0.40	517.1758	-0.0019	N	Isoflavonoid	518.106045	$C_{24}H_{22}O_{13}$	+	+	+	+	+	159, 133, 107	[7]	[7]
Genistein 7-*O*-apiofuranosyl-(1→6)-glucoside	0.56	563.1813	0.0008	N	Isoflavonoid	564.14791	$C_{26}H_{28}O_{14}$	-	+	+	-	+	133, 107	[7]	[7]
Glabridin	0.60	323.1342	0.0006	N	Isoflavonoid	324.13616	$C_{20}H_{20}O_4$	+	+	+	+	+	203, 133, 132, 189	[7]	[7]
Genistin	0.60	431.1223	-0.0019	N	Isoflavonoid	432.105649	$C_{21}H_{20}O_{10}$	+	+	+	+	+	159, 133, 107	[7]	[7]
Syringaresinol	1.06	417.1025	-0.0012	N	Lignan	418.16277	$C_{22}H_{26}O_8$	+	+	+	+	+	369, 265, 181, 179	[7]	[7]
Secoisolariciresinol diglucoside I+NH4 (SDG)	3.77	704.3402	0.0009	P	Lignan	686.27859	$C_{32}H_{46}O_{16}$	+	+	+	+	+	541, 361, 219, 179	[7]	[7]
Secoisolariciresinol diglucoside II+NH4 (SDG)	4.11	704.4017	-0.0034	P	Lignan	686.27859	$C_{32}H_{46}O_{16}$	+	+	+	+	+	541, 361, 179	[7]	[7]
Caffeic acid derivative	0.42	377.0848	-0.0002	N	Phenolic acid derivative	378.095085	$C_{18}H_{18}O_9$	+	+	+	+	+	341, 191, 181, 179, 161	[7]	[7]
sn-Glycero-3-phosphocholine	0.42	258.1099	-0.0002	P	Phospholipid	257.102827	$C_8H_{20}NO_6P$	+	+	+	+	+	257	Sirus	[8]
PI(18:3/0:0)	4.68	593.2727	-0.0028	N	Phospholipid	594.280518	$C_{27}H_{47}O_{12}P$	+	+	+	+	+	283, 255	Sirus	[8]
LysoPE(0:0/18:3)	4.68	474.2622	-0.0023	N	Phospholipid	475.269891	$C_{23}H_{42}NO_7P$	+	+	+	+	+	277, 141	Sirus	[8]

Identification	Retention time (min)	*m/z*	Error (ppm)	Mode	Chemical class	Exact Mass	MF	FB	Bp	KB	RL	CP	Mass fragments	Ref.	Reported
LysoPC(18:3)	4.79	518.3242	0.0011	P	Phospholipid	517.316842	$C_{26}H_{48}NO_7P$	+	+	-	+	+	277, 141	Sirus	[8]
LPE(18:2/0:0)	4.84	478.293	0.0002	P	Phospholipid	477.285541	$C_{23}H_{44}NO_7P$	-	-	+	-	-	281, 141	Sirus	[8]
PI(18:2/0:0)	4.87	595.2884	-0.0027	N	Phospholipid	594.280518	$C_{27}H_{47}O_{12}P$	+	-	+	+	-	281	Sirus	[8]
LysoPE(0:0/16:0)	4.93	452.2776	0.0007	N	Phospholipid	453.285541	$C_{21}H_{44}NO_7P$	-	-	+	-	-	255	Sirus	[8]
LysoPI(16:0/0:0)	4.96	571.2885	0.0006	N	Phospholipid	572.296168	$C_{25}H_{49}O_{12}P$	+	-	-	-	-	255, 141	Sirus	[8]
LysoPC(18:2)	4.98	520.3399	0.0001	P	Phospholipid	519.332492	$C_{26}H_{50}NO_7P$	+	+	+	+	-	281, 125	Sirus	[8]
LysoPE(0:0/20:2)	4.98	504.3088	-0.0007	N	Phospholipid	505.316841	$C_{25}H_{48}NO_7P$	-	-	+	-	-	307, 125	Sirus	[8]
PE(18:1/0:0)	5.04	478.293	0.0007	N	Phospholipid	479.301192	$C_{23}H_{46}NO_7P$	-	+	-	-	+	281	Sirus	[8]
PC(0:0/16:0)	5.08	496.34	0.0002	P	Phospholipid	495.332492	$C_{24}H_{50}NO_7P$	+	+	+	-	-	255, 125	Sirus	[8]
PI(18:1/0:0)	5.09	597.3028	-0.0016	N	Phospholipid	596.296168	$C_{27}H_{49}O_{12}P$	-	+	+	-	-	281, 141	Sirus	[8]
LysoPG(18:2)	5.17	507.2718	0.0010	N	Phospholipid	508.280123	$C_{24}H_{45}O_9P$	-	+	+	-	+	281, 141, 125	Sirus	[8]
LysoPC(18:1)	5.20	522.3555	0.0001	P	Phospholipid	521.348142	$C_{26}H_{52}NO_7P$	+	-	-	-	-	281, 255, 125	Sirus	[8]
LysoPE(0:0/20:1)	5.21	506.3241	-0.0011	N	Phospholipid	507.332492	$C_{25}H_{50}NO_7P$	-	+	-	-	-	309, 255, 125	Sirus	[8]

Identification	Retention time (min)	*m/z*	Error (ppm)	Mode	Chemical class	Exact Mass	MF	FB	Bp	KB	RL	CP	Mass fragments	Ref.	Reported
LysoPG(16:0/0:0)	5.28	483.2717	-0.0012	N	Phospholipid	484.280123	$C_{22}H_{45}O_9P$	+	-	+	+	-	281, 141, 125	Sirus	[8]
LPA(18:2/0:0)	5.35	433.2346	-0.0014	N	Phospholipid	434.243343	$C_{21}H_{39}O_7P$	-	-	+	-	+	281, 125	Sirus	[8]
LPC(18:0)	5.51	524.371	0.0016	P	Phospholipid	523.363792	$C_{26}H_{54}NO_7P$	+	+	+	-	-	257, 255, 125	Sirus	[8]
PI(16:1/18:2)	5.78	831.5008	-0.0018	N	Phospholipid	832.510183	$C_{43}H_{77}O_{13}P$	-	+	-	-	-	255, 125	Sirus	[8]
PC(18:2/18:3)+Na	5.88	802.5354	-0.0024	P	Phospholipid	779.546507	$C_{44}H_{78}NO_8P$	-	+	+	-	-	277, 253, 125	Sirus	[8]
PI(16:1/18:1)	5.98	833.5165	-0.0020	N	Phospholipid	834.525832	$C_{43}H_{79}O_{13}P$	-	-	-	-	+	255, 141, 225	Sirus	[8]
PC(14:0/20:2)+Na	6.06	780.5525	0.0014	P	Phospholipid	757.562157	$C_{42}H_{80}NO_8P$	-	+	+	+	-	283, 255, 141	Sirus	[8]
PI(16:3/18:0)	6.07	831.5015	-0.0020	N	Phospholipid	832.510183	$C_{43}H_{77}O_{13}P$	+	+	+	+	+	281, 125	Sirus	[8]
PI(16:0/18:2)	6.11	833.5163	-0.0018	N	Phospholipid	834.525832	$C_{43}H_{79}O_{13}P$	+	+	+	+	+	255, 125	Sirus	[8]
PC(16:1/20:1)+NH4	6.13	808.5817	-0.0025	P	Phospholipid	785.593457	$C_{44}H_{84}NO_8P$	-	+	+	+	-	253, 125	Sirus	[8]
PC(16:1/20:2)+Formate	6.20	828.5732	0.0010	N	Phospholipid	783.577807	$C_{44}H_{82}NO_8P$	+	-	-	-	-	307, 253	Sirus	[8]
PC(18:2/18:1)+Na	6.354	806.5657	-0.0002	P	Phospholipid	783.577807	$C_{44}H_{82}NO_8P$	+	+	+	+	+	283	Sirus	[8]
PC(18:2/18:2)+Formate	6.43	830.5878	-0.0012	N	Phospholipid	781.562157	$C_{44}H_{80}NO_8P$	-	-	-	-	+	281, 125	Sirus	[8]
PE(18:4/18:0)	6.49	738.5048	-0.0015	N	Phospholipid	739.515207	$C_{41}H_{74}NO_8P$	+	-	+	-	-	255	Sirus	[8]

Identification	Retention time (min)	*m/z*	Error (ppm)	Mode	Chemical class	Exact Mass	MF	FB	Bp	KB	RL	CP	Mass fragments	Ref.	Reported
PC(18:1/18:3)+Formate	6.51	826.5576	-0.0005	N	Phospholipid	781.562157	$C_{44}H_{80}NO_8P$	+	-	+	-	+	281, 125	Sirus	[8]
PC(16:0/18:3)+Na	6.59	778.5353	0.0006	P	Phospholipid	755.546507	$C_{42}H_{78}NO_8P$	-	-	-	+	-	255	Sirus	[8]
PC(20:3/16:0)	6.65	784.5852	0.0017	P	Phospholipid	783.577807	$C_{44}H_{82}NO_8P$	-	-	-	-	-	255, 125	Sirus	[8]
PC(18:3/18:1)	6.66	782.5694	-0.0027	P	Phospholipid	781.562157	$C_{44}H_{80}NO_8P$	-	-	-	+	-	281, 125	Sirus	[8]
PC(20:2/14:1)	6.70	756.5524	-0.0014	P	Phospholipid	755.546507	$C_{42}H_{78}NO_8P$	-	-	-	-	-	277, 125	Sirus	[8]
HexCer(18:1;2O/16:0;O)	6.73	714.5493	-0.0016	N	Sphingolipid	715.559834	$C_{40}H_{77}NO_9$	+	+	+	+	+	257, 125	Sirus	[8]
PE(16:1/20:5)	6.76	734.4732	-0.0031	N	Phospholipid	735.483907	$C_{41}H_{70}NO_8P$	+	+	+	+	-	277, 125	Sirus	[8]
PI(18:1/16:0)	6.81	835.5301	-0.0006	N	Phospholipid	836.541483	$C_{43}H_{81}O_{13}P$	+	+	+	+	-	279, 125	Sirus	[8]
2,3-Dilinoleoyl-sn-glycerol	6.89	617.513	-0.0010	P	Glycerolipid	616.506675	$C_{39}H_{68}O_5$	+	-	+	-	-	255, 125	Sirus	[8]
PS(20:1/18:2)	6.89	814.5586	-0.0034	P	Phospholipid	813.551987	$C_{44}H_{80}NO_{10}P$	+	+	+	+	+	307, 255	Sirus	[8]
PE(16:1/18:1)	6.97	714.5029	-0.0019	N	Phospholipid	715.515207	$C_{39}H_{74}NO_8P$	+	-	+	+	-	255, 125	Sirus	[8]
PE(18:2/18:3)	6.98	736.4905	-0.0006	N	Phospholipid	737.499557	$C_{41}H_{72}NO_8P$	+	-	-	+	-	257	Sirus	[8]
PE(18:2/16:0)+Na	6.98	738.506	0.0019	P	Phospholipid	715.515207	$C_{39}H_{74}NO_8P$	+	-	+	+	-	255, 125	Sirus	[8]

Identification	Retention time (min)	*m/z*	Error (ppm)	Mode	Chemical class	Exact Mass	MF	FB	Bp	KB	RL	CP	Mass fragments	Ref.	Reported
PI(18:0/16:1)	6.99	835.5313	-0.0018	N	Phospholipid	834.525832	$C_{43}H_{79}O_{13}P$	+	+	+	+	+	253, 281	Sirus	[8]
PI(16:0/18:3)	7.07	831.5006	0.0012	N	Phospholipid	832.510183	$C_{43}H_{77}O_{13}P$	+	+	+	+	+	255, 125	Sirus	[8]
PG(16:0/18:2)	7.11	745.5002	-0.0014	N	Phospholipid	744.494138	$C_{40}H_{73}O_{10}P$	+	+	+	+	+	279, 255, 125	Sirus	[8]
PE(14:0/20:3)	7.22	712.4912	0.0010	N	Phospholipid	713.499557	$C_{39}H_{72}NO_8P$	+	+	+	+	+	305	Sirus	[8]
PE(18:2/18:2)	7.23	738.5063	-0.0015	N	Phospholipid	739.515207	$C_{41}H_{74}NO_8P$	+	+	+	+	+	381, 283	Sirus	[8]
PC(22:1/14:1)	7.24	786.6003	-0.0007	P	Phospholipid	785.593457	$C_{44}H_{84}NO_8P$	+	+	+	+	+	225	Sirus	[8]
PC(14:0/20:1)	7.25	760.5836	-0.0029	P	Phospholipid	759.577807	$C_{42}H_{82}NO_8P$	+	+	+	+	+	305, 223	Sirus	[8]
PC(18:0/14:0)	7.25	734.5686	-0.0037	P	Phospholipid	733.562157	$C_{40}H_{80}NO_8P$	+	+	+	+	+	255, 125	Sirus	[8]
PS(22:2/16:0)	7.306	816.5743	-0.0013	P	Phospholipid	815.567637	$C_{44}H_{82}NO_{10}P$	-	+	+	-	+	383, 255	Sirus	[8]
MGDG(18:1/18:3)+Na	7.38	801.5541	0.0016	P	Glycolipid	778.5595	$C_{45}H_{78}O_{10}$	+	-	+	+	+	283	Sirus	[8]
PC(20:2/14:0)	7.53	758.5698	-0.0008	P	Phospholipid	757.5621551	$C_{42}H_{80}NO_8P$	+	-	-	+	-	223	Sirus	[8]
PI(16:2/18:1)	7.53	831.5013	-0.0006	N	Phospholipid	832.510183	$C_{43}H_{77}O_{13}P$	+	+	+	+	-	281, 125	Sirus	[8]
PC(18:1/18:1)	7.53	786.5995	-0.0036	P	Phospholipid	785.593457	$C_{44}H_{84}NO_8P$	-	-	+	-	-	281, 141	Sirus	[8]

Identification	Retention time (min)	*m/z*	Error (ppm)	Mode	Chemical class	Exact Mass	MF	FB	Bp	KB	RL	CP	Mass fragments	Ref.	Reported
PE (16:0/18:2)	7.56	714.5049	-0.0019	N	Phospholipid	715.515207	$C_{39}H_{74}NO_8P$	-	-	-	-	-	255, 125	Sirus	[8]
PE(18:2/16:0)	7.58	716.5211	0.0021	P	Phospholipid	715.515207	$C_{39}H_{74}NO_8P$	+	+	-	-	-	257, 255, 125	Sirus	[8]
PC(18:3/18:0)	7.59	784.5833	-0.0020	P	Phospholipid	783.577807	$C_{44}H_{82}NO_8P$	-	+	-	-	+	281	Sirus	[8]
PE(18:2/18:1)	7.63	740.5201	-0.0013	N	Phospholipid	741.530857	$C_{41}H_{76}NO_8P$	-	-	+	-	-	281, 141	Sirus	[8]
PC(18:3/16:0) +Na	7.77	778.538	0.0006	P	Phospholipid	755.546507	$C_{42}H_{78}NO_8P$	-	-	+	-	-	277, 125	Sirus	[8]
PE(18:1/18:2)	8.99	740.5221	-0.0017	N	Phospholipid	741.530857	$C_{41}H_{76}NO_8P$	+	+	-	+	-	283, 255, 125	Sirus	[8]
Procyanidin C1	0.37	867.2372	0.0008	P	Procyanidin	866.20582	$C_{45}H_{38}O_{18}$	+	-	-	+	-	289	[5]	[5]
Phaseoside IV	2.24	925.2586	0.0025	P	Saponin	924.508255	$C_{48}H_{76}O_{17}$	-	+	+	-	+	779 617, 599, 441	[7]	[8]
Soyasapogenol C	3.316	441.3724	0.0011	P	Saponin	440.36543	$C_{30}H_{48}O_2$	-	-	+	-	-	405, 205, 143	[7]	[8]
Phaseoside I	3.34	1251.598	-0.0019	N	Saponin	1252.60882	$C_{59}H_{96}O_{28}$	+	+	+	-	-	779 617, 599, 441	[7]	[8]
Soyasaponin Bd	4.15	957.5058	0.0034	N	Saponin	956.498085	$C_{48}H_{76}O_{19}$	-	+	+	-	+	613, 441	[7]	[8]
Soyasaponin Be	4.16	939.4931	0.0005	N	Saponin	940.50317	$C_{48}H_{76}O_{18}$	+	-	-	+	-	793, 613, 423, 441	[7]	[8]

Identification	Retention time (min)	*m/z*	Error (ppm)	Mode	Chemical class	Exact Mass	MF	FB	Bp	KB	RL	CP	Mass fragments	Ref.	Reported
Soyasaponin Bb/Soyasaponin I	4.21	943.5256	-0.0037	P	Saponin	942.51882	$C_{48}H_{78}O_{18}$	+	+	+	+	-	795, 615, 457	[7]	[8]
Soyasaponin III	4.23	797.4675	0.0038	P	Saponin	796.46091	$C_{42}H_{68}O_{14}$	-	+	-	-	+	615, 405457, 441	[7]	[8]
Kaikasaponin III	4.32	925.5165	-0.0001	N	Saponin	926.523905	$C_{48}H_{78}O_{17}$	+	+	+	+	+	779, 617, 599, 441	[7]	[8]
Soyasaponin alphag II	4.4059	1083.54	0.0014	N	Saponin	1084.54543	$C_{54}H_{84}O_{22}$	+	+	+	+	+	897, 595, 571, 405	[7]	[8]
Dehydrosoyasaponin I	4.40	939.4952	-0.0040	N	Saponin	940.50317	$C_{48}H_{76}O_{18}$	+	+	+	-	+	613, 455	[7]	[8]
Soyasaponin alphag I	4.46	1067.543	0.0032	N	Saponin	1068.550515	$C_{54}H_{84}O_{21}$	+	+	+	-	+	879, 733, 205	[7]	[8]
Soyasapogenol E	5.90	455.3513	-0.0015	N	Saponin	456.360345	$C_{30}H_{48}O_{3}$	+	+	+	+	+	441, 405, 205, 143	[7]	[8]
Soyacerebroside I+Na	6.63	736.5307	-.0033	P	Saponin	713.544184	$C_{40}H_{75}NO_{9}$	-	-	-	+	-	441	[7]	[8]
Dihydrosoyasaponin Ba	6.69	961.5846	0.0031	P	Saponin	960.529385	$C_{48}H_{80}O_{19}$	-	-	-	-	-	595, 423, 441	[7]	[8]
Soyasaponin II	9.06	913.6886	0.0015	P	Saponin	912.508255	$C_{47}H_{76}O_{17}$	-	+	-	-	-	893, 615, 457	[7]	[8]
Oleuropein aglycon	0.91	377.0841	-0.0018	N	Seco-iridoid	378.13147	$C_{19}H_{22}O_{8}$	+	+	-	+	-	539, 345, 307, 275	[9]	[9]
Oleuropein	0.42	539.1375	-0.0007	N	Seco-iridoid	540.184295	$C_{25}H_{32}O_{13}$	+	-	+	+	-	345, 307,	[9]	[9]
Dihydro-dihydroxy megastigmadien-one-[apiosyl-glucoside]	5.40	519.2614	0.0012	N	Terpene	518.23633	$C_{24}H_{38}O_{12}$	+	+	+	-	+	387	[4]	[4]

Identification	Retention time (min)	*m/z*	Error (ppm)	Mode	Chemical class	Exact Mass	MF	FB	Bp	KB	RL	CP	Mass fragments	Ref.	Reported
Coumesterol acetate	5.49	325.1832	0.0001	N	Sterol	326.042654	$C_{17}H_{10}O_7$	+	+	-	+	-	265	[4]	[4]
β-Sitosterol 3-O-glucoside+Formate	6.42	621.4345	0.0030	N	Sterol	576.43899	$C_{35}H_{60}O_6$	-	+	+	-	+	413	HMDB	[10]
Stigmastenone	6.95	413.2664	0.0011	P	Sterol	412.370515	$C_{29}H_{48}O$	-	+	+	+	+	395	Sirus	[11]
Piceid	4.43	389.1972	0.0005	N	Stilbene	390.13147	$C_{20}H_{22}O_8$	+	-	+	-	-	211	[4]	[4]
Gibberellin A8	0.46	365.1053	-0.0014	P	Terpene	364.152205	$C_{19}H_{24}O_7$	-	-	+	-	-	317	[4]	[4]

Note: N; negative ion mode, P; positive ion mode, HMDB: (HMDB) (www.hmdb.ca).
* Reported before in legume species.

Table S2. The metabolic pathways covered by the identified metabolites in the five legume samples.

Identified KEGG Pathway	Total compounds	Hits	Raw p	-log10(p)	Holm adjust	FDR	Impact
alpha-Linolenic acid metabolism	28	3	3.92E-11	1.04E+01	1.02E-09	1.02E-09	0.11
Glycine, serine and threonine metabolism	33	1	6.36E-10	9.20E+00	1.59E-08	8.27E-09	0.00
Biosynthesis of unsaturated fatty acids	22	2	1.09E-07	6.96E+00	2.61E-06	9.42E-07	0.00
Flavone and flavonol biosynthesis	47	2	7.55E-07	6.12E+00	1.74E-05	4.91E-06	0.00
Diterpenoid biosynthesis	28	1	4.54E-06	5.34E+00	1.00E-04	2.36E-05	0.01
Glycerophospholipid metabolism	37	6	1.50E-05	4.83E+00	3.14E-04	6.48E-05	0.38
Valine, leucine and isoleucine degradation	37	1	2.29E-04	3.64E+00	4.58E-03	5.96E-04	0.00
Valine, leucine and isoleucine biosynthesis	22	1	2.29E-04	3.64E+00	4.58E-03	5.96E-04	0.00
Glucosinolate biosynthesis	65	1	2.29E-04	3.64E+00	4.58E-03	5.96E-04	0.00
Aminoacyl-tRNA biosynthesis	46	1	2.29E-04	3.64E+00	4.58E-03	5.96E-04	0.00
Linoleic acid metabolism	4	2	6.48E-04	3.19E+00	1.04E-02	1.53E-03	1.00
Galactose metabolism	27	3	7.06E-04	3.15E+00	1.06E-02	1.53E-03	0.15
Flavonoid biosynthesis	10	3	8.25E-03	2.08E+00	1.16E-01	1.65E-02	0.50
Glycerolipid metabolism	21	1	1.53E-02	1.82E+00	1.99E-01	2.65E-02	0.06
Phosphatidylinositol signaling system	26	1	1.53E-02	1.82E+00	1.99E-01	2.65E-02	0.00
Anthocyanin biosynthesis	11	1	7.18E-02	1.14E+00	7.90E-01	1.17E-01	0.33
Tyrosine metabolism	16	1	1.31E-01	8.83E-01	1.00E+00	1.89E-01	0.06
Isoquinoline alkaloid biosynthesis	6	1	1.31E-01	8.83E-01	1.00E+00	1.89E-01	0.00
Arachidonic acid metabolism	12	1	2.45E-01	6.12E-01	1.00E+00	3.19E-01	0.00
Phenylpropanoid biosynthesis	46	2	2.45E-01	6.11E-01	1.00E+00	3.19E-01	0.06
Steroid biosynthesis	45	2	3.13E-01	5.05E-01	1.00E+00	3.87E-01	0.01
Glycosylphosphatidylinositol (GPI)-anchor biosynthesis	13	1	3.70E-01	4.32E-01	1.00E+00	4.37E-01	0.00
Starch and sucrose metabolism	22	1	6.94E-01	1.59E-01	1.00E+00	7.84E-01	0.09
Arginine and proline metabolism	34	1	8.72E-01	5.94E-02	1.00E+00	8.75E-01	0.00
beta-Alanine metabolism	18	1	8.72E-01	5.94E-02	1.00E+00	8.75E-01	0.00
Lysine degradation	18	1	8.75E-01	5.79E-02	1.00E+00	8.75E-01	0.00

Note: Total is the total number of compounds in the pathway; the Hits is the matched number from the user uploaded data; the Raw p is the original p value calculated from the enrichment analysis; the Holm p is the p value adjusted by Holm-Bonferroni method; the FDR p is the p value adjusted using False Discovery Rate; the Impact is the pathway impact value calculated from pathway topology analysis.

Table S3. Docking scores of positively correlated metabolites.

Comp.	AChE	BuChE
(+)-Catechin 3-O-glucoside	-6.80139	-8.08223
β-Chaconine	-7.86221	-10.328
Dehydro-Soyasaponin I	-6.37957	-11.4179
Ferutinine	-6.54977	-7.78641
Genistein 7-*O*-apiofuranosyl-(1→6)-glucoside	-9.47558	-8.86934
Genistin	-8.43443	-7.34533
Kaempferol-3-O-[6''-malonyl-apiofuranosyl-(1→2)- glucoside]	-8.19725	-9.39954
Kaempferol-3-*O*-(2''-*O*-glucosyl)-rutinoside	-8.3306	-10.3045
Kaikasaponin III	-5.77688	-11.36
N, N'-Diferuloyl putrescine	-9.57584	-8.41892
Oleuropein	-9.28246	-9.25216
Phaseoside I	-4.51652	-12.24
Prodelphinidin B3	-7.10694	-9.63155
Quercetin 3,4'-*O*-diglucoside	-8.75627	-9.78677
Sissotrin	-9.00546	-8.00212
Solanidine	-5.73269	-7.73354
Soyasapogenol E	-5.78748	-7.94037
Soyasaponin alphag	-0.37172	-10.2818
Co-crystalized protein	-9.51489	-8.89345

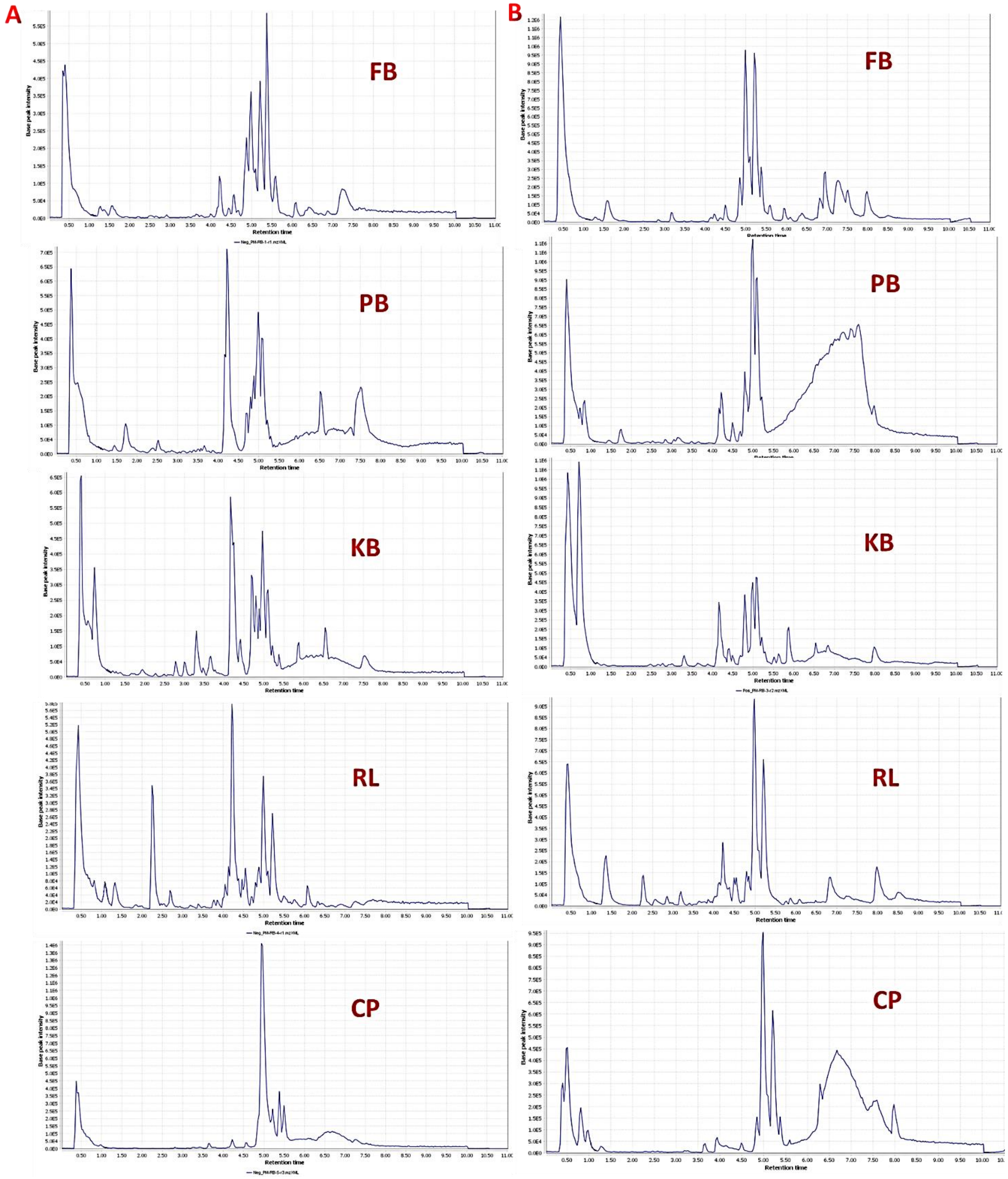

Fig. S1. The representative base peak chromatograms of the five legumes in **A)** negative and **B)** positive ionization modes.

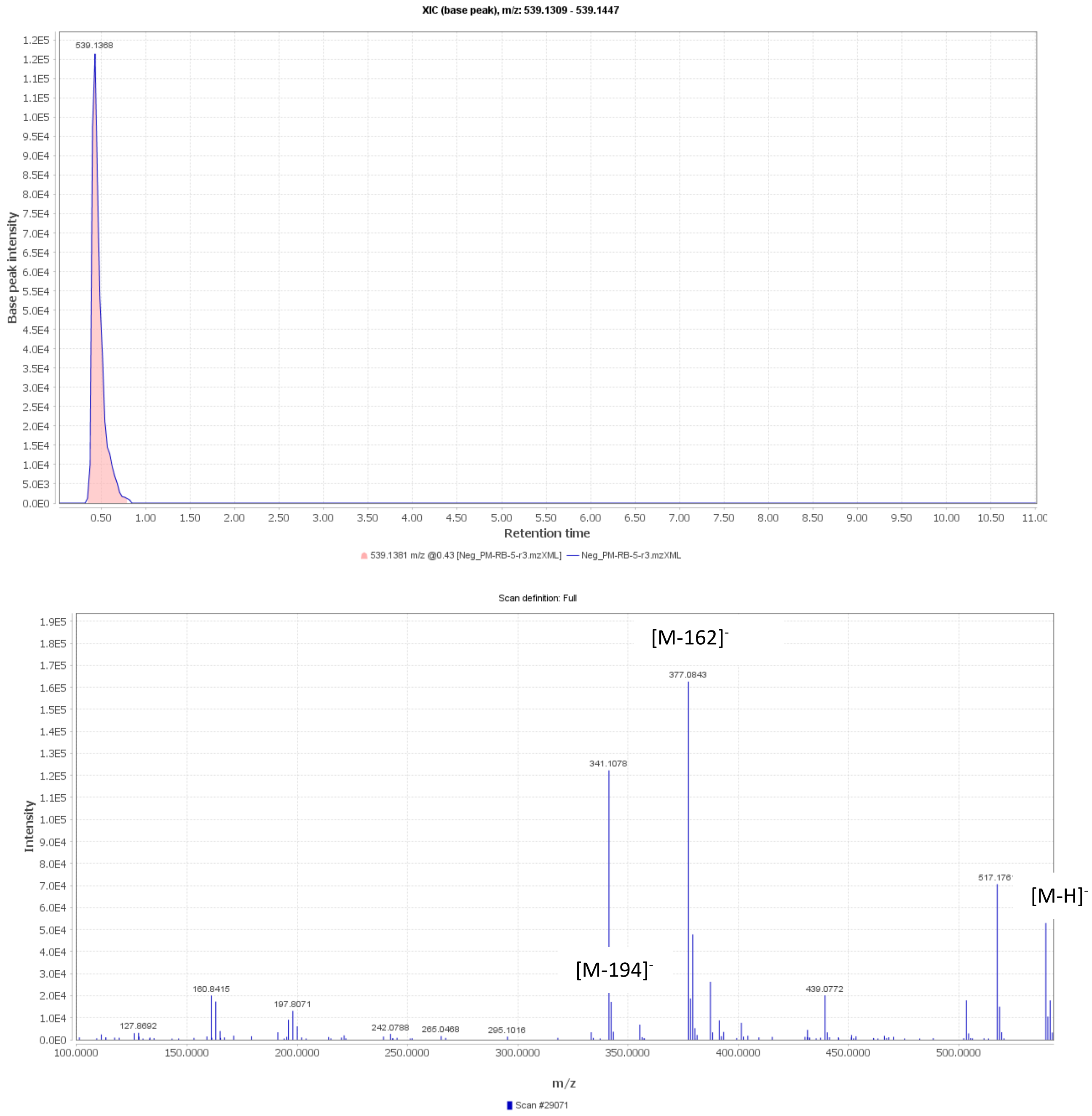

Fig. S2. The chromatograms of the identification of oleuropein in the negative ionization mode.

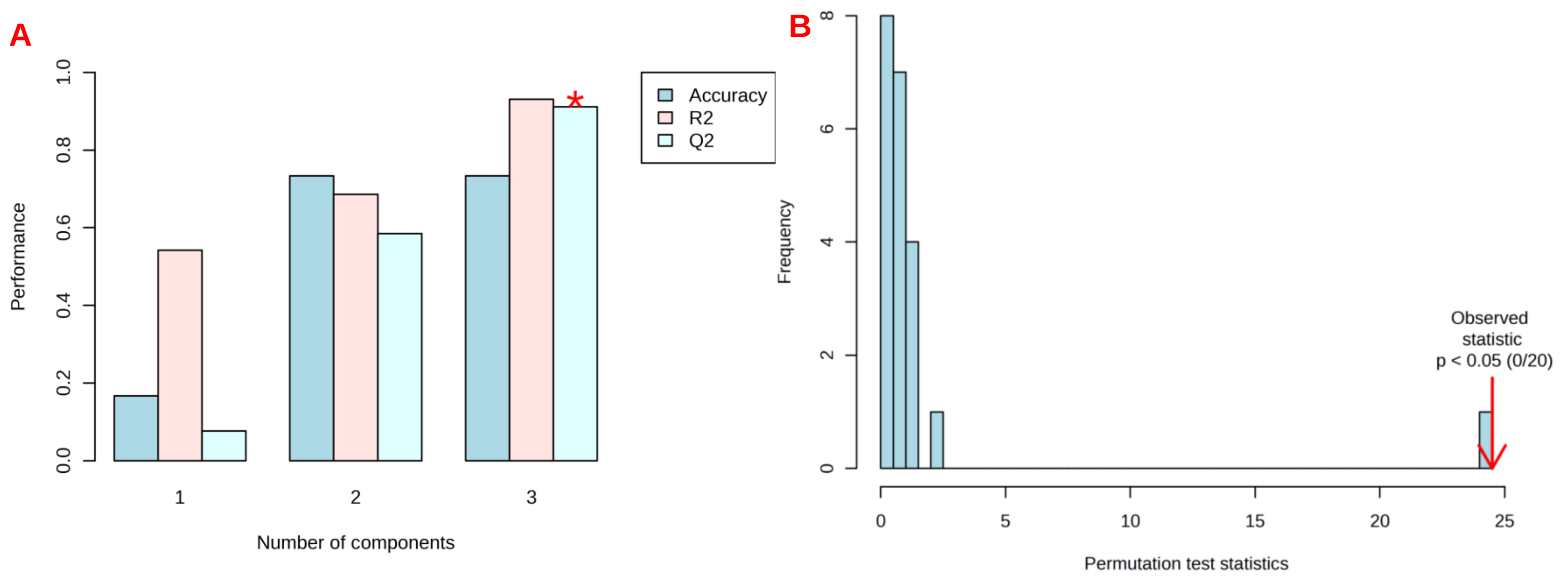

Fig. S3. 5-Fold cross-validation (A) and permutation test (B) describing the R2 and Q2 of the PLS-DA model for the identified metabolites in the five legumes.

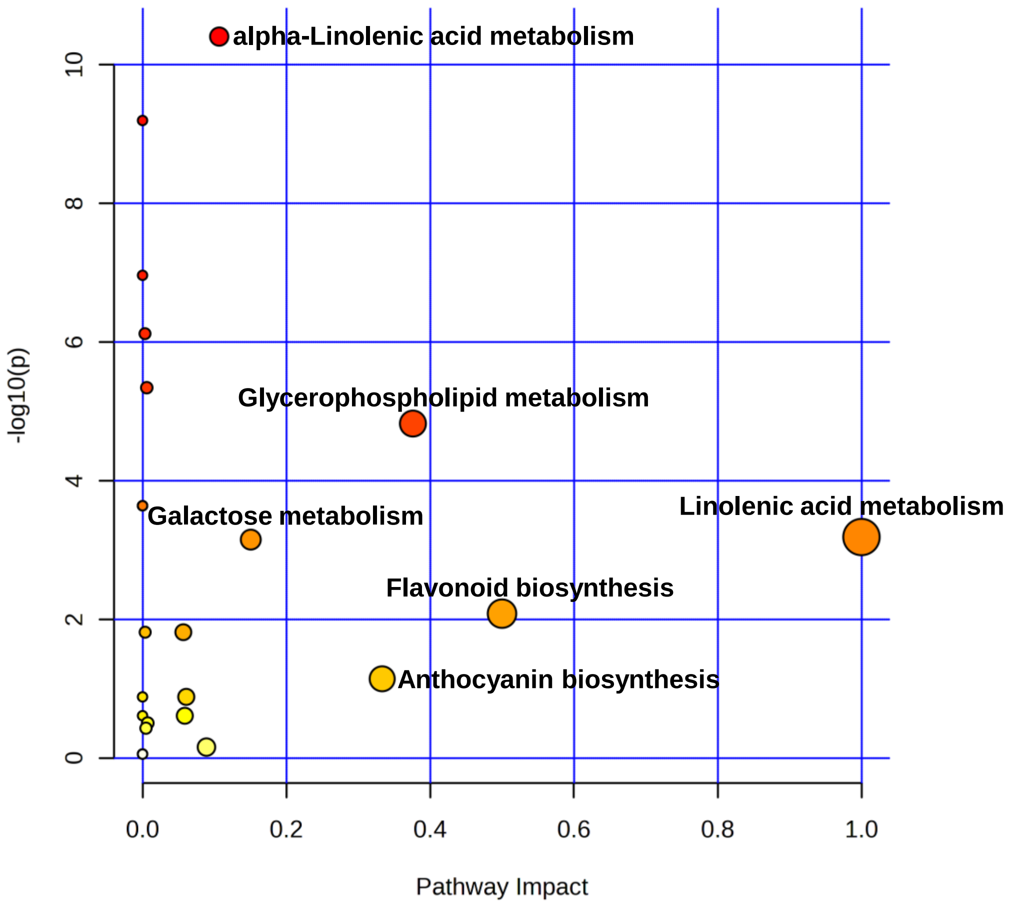

Fig. S4. Metabolome map of significant metabolic pathways detected by KEGG in the five analyzed legumes. Significantly changed pathways according to *p*-values from the pathway enrichment analysis (*y*-axis) and pathway impact values from the pathway topology analysis (*x*-axis) are shown. Large sizes and dark colors represent major pathway enrichment and high pathway impact values, respectively.

References

[1] Nielsen, S. D., Schmidt, J. M., Kristiansen, G. H., Dalsgaard, T. K. & Larsen, L. B. Liquid chromatography mass spectrometry quantification of α-solanine, α-chaconine, and solanidine in potato protein isolates. *Foods* **9**, 416 (2020).

[2] Saied, D. B. & Farag, M. A. How does maturity stage affect seeds metabolome via UPLC/MS based molecular networking and chemometrics and in relation to antioxidant effect? a case study in 4 major cereals and legumes. *Food Chem.* **426**, 136491 (2023).

[3] Giusti, F. *et al.* Analysis of 17 polyphenolic compounds in organic and conventional legumes by high-performance liquid chromatography-diode array detection (HPLC-DAD) and evaluation of their antioxidant activity. *Inte J Food Sci Nutr* **69**, 557-565 (2018).

[4] Llorach, R. *et al.* Comparative metabolite fingerprinting of legumes using LC-MS-based untargeted metabolomics. *Food Res Inter***126**, 108666 (2019).

[5] Zhang, B. *et al.* Phenolic profiles of 20 Canadian lentil cultivars and their contribution to antioxidant activity and inhibitory effects on α-glucosidase and pancreatic lipase. *Food Chem.* **172**, 862-872 (2015).

[6] Caprioli, G. *et al.* Lipid nutritional value of legumes: Evaluation of different extraction methods and determination of fatty acid composition. *Food Chem.***192**, 965-971 (2016).

[7] Mekky, R. H. *et al.* Profiling of phenolic and other compounds from Egyptian cultivars of chickpea (*Cicer arietinum* L.) and antioxidant activity: A comparative study. *RSC Adv* **5**, 17751-17767 (2015).

[8] Moreau, R. A. *et al.* Phytosterols and their derivatives: Structural diversity, distribution, metabolism, analysis, and health-promoting uses. *Progress in Lipid Res.* **70**, 35-61 (2018).

[9] Alu'datt, M. H. *et al.* Current perspectives on fenugreek bioactive compounds and their potential impact on human health: A review of recent insights into functional foods and other high value applications. *J Food Sci* (2024).

[10] Mustafa, A. M. *et al.* Polyphenols, saponins and phytosterols in lentils and their health benefits: an overview. *Pharmaceuticals* **15**, 1225 (2022).

[11] HASHIM, N. B. MICROWAVE ASSISTED EXTRACTION (MAE) OF BETA-SITOSTEROL FROM PLANT LEGUMES POD.
